# Addressing the Needs of Breast Cancer Survivors

**DOI:** 10.1007/s11912-025-01687-x

**Published:** 2025-06-13

**Authors:** Humaira Sarfraz, Fatima Tuz Zahra, Utsav Joshi, Tracey O’Connor

**Affiliations:** 1 University of Alabama at Birmingham, ONeal Comprehensive Cancer Center NP2512 1720 2 Avenue South, Birmingham, AL 35294-3300 USA; 2 Moffitt Cancer Center, 12902 USF Magnolia Drive, Tampa, FL 33612 USA

**Keywords:** Breast cancer, Survivorship

## Abstract

**Introduction:**

Breast cancer survivors experience unique treatment-related side effects that have long-standing and life-altering implications. These remain a challenging aspect both in terms of diagnosis and management for these patients.

**Objective:**

This article is a review of the latest key literature in the management of common side effects of treatment in breast cancer survivors namely infertility, vasomotor symptoms, sexual dysfunction, peripheral neuropathy, lymphedema, musculoskeletal symptoms, anthracycline-related cardiotoxicity, secondary malignancies, role of ctDNA, lifestyle modifications, strategies to maintain bone mineral density and addressing fear of cancer recurrence.

**Results:**

Recent research in the field has led to novel advancements for survivors planning a family, pharmacotherapeutic options for hot flashes, overcoming barriers such as adherence to therapy and management of long-term side effects, and a incorporating a multimodal approach to improve sexual health in women, neuropathy, musculoskeletal issues and lymphedema.

**Summary:**

There have been significant strides in the understanding and management of issues in breast cancer survivors. The increasing awareness and multimodal approach will continue to improve outcomes for survivors.

## Introduction

Given the advances in early cancer detection and optimal integration of local and novel systemic therapeutic options, early-stage breast cancer has become a largely curable disease and outcomes have significantly improved. [1, 3] This has resulted in a higher prevalence of breast cancer survivors who are affected by chronic treatment-related side effects highlighting the importance of survivorship care. [4] Breast cancer survivors encounter unique challenges, including physical and psychosocial issues as sequelae of cancer treatment, pre-existing chronic conditions, risk of cancer recurrence, and secondary malignancies. These can negatively affect the survivor’s quality of life, physical function, adherence to adjuvant treatment and surveillance. In this review article, we will focus on some key areas such as fertility issues, vasomotor symptoms, sexual dysfunction, neuropathy, lymphedema, anthracycline-related cardiotoxicity, secondary malignancies, role of ctDNA, lifestyle modifications, strategies to maintain bone mineral density and addressing fear of cancer recurrence.

### Infertility

While breast cancer is the most prevalent malignancy in women, almost 10% of newly identified cases involve women in the reproductive age group. [5] For many of these young women diagnosed with breast cancer, fertility preservation and childbearing hold great significance. [6] Many of these survivors have not completed their families at the time of cancer detection. Helping Ourselves-Helping Others (HOHO), the Young Women's BC Study and European HOHO cohort, led by the International Breast Cancer Study Group (IBCSG) showed that over half of young patients with breast cancer were concerned regarding fertility [7, 8] The risk of infertility may negatively impact treatment decisions and subsequent outcomes for cancer patients as some patients may refrain from recommended therapies. [7, 9] The current standard of care for early breast cancer often involves systemic therapies such as highly gonadotoxic chemotherapy. Meanwhile the reproductive risk with other therapies such as immunotherapy, endocrine therapy, and targeted agents such as Her2 directed therapies, poly (ADP-ribose) polymerase (PARP) inhibitors, and CDK 4/6 inhibitors is largely unexplored. Patients treated with chemotherapy are at risk for developing premature ovarian failure (POI) and subsequent infertility. [10] All International guidelines recommend oncofertility counseling prior to treatment. All patients interested in having a future family should be referred to a fertility specialist. [11, 13].

Fertility preservation techniques include cryopreservation of oocytes/embryos with controlled ovarian stimulation (COS). Alternatively, ovarian tissue cryopreservation is an option for women who are unable or who should not delay gonadotoxic treatment due to aggressive malignancy. [12] Studies show that COS was not associated with inferior results for recurrence risk (RR 0.58, 95% CI 0.46–0.76), event-free survival (EFS, HR 0.76, 95% CI 0.55–1.06), or mortality (RR 0.54, 95% CI 0.38–0.76).

Gonadotropin-releasing hormone agonist initiation in premenopausal women at least one week before cytotoxic chemotherapy reduces POI risk and potentially decreases subsequent infertility regardless of the estrogen receptor status for breast cancer. [14] A meta-analysis demonstrated that risk was significantly lowered with concurrent GnRH agonist and chemotherapy (POI rates 30.9% versus 14.1%, adjusted OR 0.38; 95% CI 0.26–0.57; P 0.001). [15] The gonadotrophic treatment causes the ovaries to become quiescent, and this appears to decrease the risk of chemotherapy-induced POI and may increase the chance of pregnancy following treatment conclusion. Therefore, this should be a consideration for both hormone receptor-positive and negative breast cancer patients. However, this is recommended to be utilized in conjunction with, but not as a substitute for, other modalities of family planning. Also, it is worth noting that GnRH treatment delays the onset of menopause by preserving ovarian function.

There is frequent concern among survivors that pregnancy after a cancer diagnosis may increase the risk of breast cancer recurrence. However, retrospective studies have not demonstrated worsening of disease outcomes with a subsequent pregnancy. [16, 18] Adjuvant endocrine therapy for 5–10 years substantially decreases the risk of cancer recurrence in patients with hormone positive disease but during this period ovarian reserve decreases and pregnancy is contraindicated. [19] Recent studies show that even for women with hormone receptor-positive breast cancer, pregnancy after appropriate cancer treatment and follow-up has no safety implications for the mother or baby, although longer follow up is needed. [18, 20] In the recently reported POSITIVE trial, women less than 43 years of age (mostly stage I and II) who completed 18–30 months of endocrine therapy and wanted to become pregnant held endocrine therapy for up to 2 years. These patients then resumed endocrine therapy to complete 5–10 years of treatment after pregnancy and breastfeeding or following an unsuccessful trial of conception. The study showed no increase in short-term risk of breast cancer events and temporary interruption of endocrine therapy may be incorporated as a safe option in the appropriate patient population. [21, 22] Longer follow-up data from this trial will be critical in determining the optimal approach for patients on endocrine therapy who wish to become pregnant. Following the diagnosis of cancer, the live birth rate after embryo cryopreservation is estimated at 40–50% while after oocyte preservation ranges between 30–40% per transfer. [23].

Women with hereditary breast and ovarian syndromes are recommended to undergo risk-reducing gynecological surgeries at an early age adding to the already complex decision-making process for these patients. [24] Risk-reducing bilateral salpingo-oophorectomy is recommended between 35–40 years of age for BRCA 1 carriers and 40–45 years for BRCA 2 carriers or once their family is completed. A recent global study shows that risk-reducing bilateral mastectomy and salpingo-oophorectomy improved overall survival outcomes among young woman who have a previous history of early-onset breast cancer and who carry the BRCA mutation. [6] [25] Preliminary data shows reduced ovarian reserve at breast cancer diagnosis in the presence of BRCA 1/2 pathogenic variants (PV) compared to women without genetic defects. [26] However, there is a paucity of data regarding the efficacy and safety of various fertility preservation options after breast cancer diagnosis in patients carrying BRCA PV [27].

Patients receiving immunotherapy are recommended to adopt birth control methods while on treatment and 5 months after completion of immunotherapy per the National Comprehensive Cancer Network guidelines. [28].

For women who experience infertility after completion of cancer treatment, other modalities of building a family including adoption, donor oocytes, and embryos may be an option. However, these are cost and time-intensive and not available as an option for many patients.

### Vasomotor Symptoms

Hot flashes are a common symptom in women on endocrine therapy and are noted to be more pronounced in younger women. These are highly prevalent in women on endocrine therapy with 80% of women on tamoxifen and 93% of those on ovarian suppression reporting significant symptoms. These can be challenging to manage and negatively impact quality of life for survivors. [29] Data shows that behavioral modifications including avoiding triggers, exercise, and dressing in layers may be effective. Based on recent randomized trials, cognitive behavioral therapy is another beneficial option for the treatment of vasomotor symptoms. [30].

Multiple pharmacologic therapies have been investigated for the management of vasomotor symptoms. Gabapentin is noted to be effective but can cause significant sedation and fluid retention as a side effect. [31] A greater than 50% decrease in vasomotor symptoms was demonstrated with serotonin receptor inhibitors (SSRI) such as paroxetine and serotonin-norepinephrine reuptake inhibitors (SNRI) including venlafaxine, and these are generally well tolerated. Drug interactions with tamoxifen must be considered when choosing an SSRI or SNRI. Tamoxifen is metabolized to its active form by the enzyme CYP2D6. It is recommended to avoid antidepressants like bupropion, paroxetine and fluoxetine given their strong inhibition of CYP2D6 and potential decreased tamoxifen efficacy with concurrent use. Other drugs such as citalopram and escitalopram are preferred alternatives when choosing an antidepressant for patients on tamoxifen. [32, 34] Transdermal clonidine was reported to cause a 37% reduction in hot flashes, but use is limited due to other more effective and better tolerated modalities of treatment. Side effects from clonidine include sleep disruption, constipation, and dry mouth. [35] Oxybutynin was noted to be effective in reducing episodes of hot flashes in perimenopausal and postmenopausal women. However, they may experience lower-grade side effects such as dry mouth, abdominal pain, and difficulty urinating. [36].

The SKYLIGHT1 study was a randomized, double-blind placebo-controlled trial that showed a significant reduction in vasomotor symptoms relating to menopause with Fezolinetant (non-hormonal Neurokinin 3 receptor antagonist). A rare but potential side effect noted on this was liver enzyme elevations but these were generally transient and mild. Liver function tests should be monitored while the patient is on Fezolinetant. [37] There is no conclusive evidence at this point regarding the effectiveness of acupuncture for the treatment of hot flashes. [38].

### Sexual Dysfunction

Sexual concerns in survivors of breast cancer can include a constellation of symptoms including vulvovaginal symptoms, decreased sexual desire, and poor body image leading to a negative impact on quality of life. [39] The cornerstone of adjuvant treatment for early-stage hormone receptor-positive breast cancer can extend for 5–10 years and includes therapies such as ovarian suppression in premenopausal women, tamoxifen, or an aromatase inhibitor. The extended period of estrogen lowering to below-normal postmenopausal levels and modulation of the estrogen receptor can lead to distressing symptoms such as vaginal dryness and painful intercourse. These undesirable toxicities of treatment are often under-recognized and not optimally addressed. The spectrum of gynecological manifestations is termed the genitourinary syndrome of menopause (GSM) and includes dryness, burning, dysuria, and itching. These symptoms may arise due to atrophic changes in the mucosa of the vagina and vulvar area. Some over-the-counter topical therapies such as alcohol-based wipes, items with artificial fragrances, parabens, propylene alcohol, and glycerin may be irritants and further exacerbate these symptoms. [29] The initial step is to reduce potential exposure to offending agents and addition of vaginal moisturization with simple emollients such as organic coconut oil and once burning symptoms lessen, hyaluronic acid-based moisturizers may be added. These are available as gels, creams, or suppositories. [40] These should be used consistently several times a week. During sexual activity, silicone or water-based lubricants are effective. Women with menopausal dyspareunia following breast cancer treatment reported decreased pain with the application of liquid lidocaine to the vulvar vestibule before penetration. [41] Also, it must be noted that generally, tamoxifen is associated with a lower risk of vaginal symptoms, vaginal atrophy, and GSM symptoms when compared to aromatase inhibitors. Therefore, a potential option for patients for patients with significant vaginal toxicity symptoms could be switching to tamoxifen.

In advanced cases, symptoms may be attributable to vaginal stenosis or shortening and the patient may not be able to engage in sexual activity. For such cases, low-dose vaginal hormones (estradiol or dehydroepiandrosterone [DHEA] have been studied. This is postulated to enhance collagen remodeling and augment elasticity and mucosal thickness. [42] In conjunction with this, vaginal dilators may be used (recommended usage is three times each week for 10 min in each session). [43].

In situations where symptoms are refractory to non-hormonal pharmacological and non-pharmacological options, vaginal estradiol can be considered an effective management option for GSM. However, there is a concern for potential systemic absorption and elevation of serum estradiol levels which raises concerns for its utilization in patients with a breast cancer history. Utilizing lower doses of commercially available vaginal estrogens and application following nonhormonal moisturizer use to allow for mucosal atrophy recovery may help mitigate the risk of absorption. [44] A large case-controlled study showed no increased risk of breast cancer recurrence with vaginal estrogen use. [45] Similarly, a large observational Danish Cohort study of postmenopausal women with early-stage hormone receptor-positive breast cancer who received systemic or local estrogen for endocrine-related genitourinary syndrome did not show an elevated recurrence risk or death. However, among patients using vaginal estrogen therapy while receiving aromatase inhibitors, there was a higher observed risk of cancer recurrence, although this did not translate into an increased risk of mortality. [46] In conclusion, there may be a potential risk of breast cancer recurrence from using vaginal estrogen; this should be carefully weighed against the risk of recurrence due to stopping endocrine therapy secondary to side effects. [47].

The NCCTG N10 C1 (Alliance) was a randomized trial showing improvement in symptoms of vaginal dryness and dyspareunia with vaginal dehydroepiandrosterone use without significantly raising serum estradiol levels. [48] The 2018 American Society of Clinical Oncology Guideline Summary recommends that patients whose symptoms do not respond to non-hormonal treatment may attempt a transient trial of vaginal DHEA or estrogen after a thorough discussion regarding risk versus benefits. [49] Additionally, a gynecologic evaluation to assess for pelvic floor spasm and vaginal stenosis should be strongly considered. Pelvic therapists often are a great resource in assisting with biofeedback, exercise, and manual therapy. The use of vaginal laser for GSM is an area with a paucity of evidence to support benefit. While some previous single-arm cohort studies and recent systemic review suggested some improvement in vaginal symptoms, a randomized double-blind clinical trial showed no significant improvement after laser therapy [50–52] Currently, there is an ongoing Alliance REVITALIZE study evaluating laser therapy for vaginal dryness and sexual dysfunction in survivors.

In summary, a multimodal approach minimizing the use of irritants, regular moisturization, using lubricants during sexual activity, utilization of resources such as pelvic floor and sexual therapists, and the judicious use of DHEA or low dose vaginal estrogen can help address this issue. [53].

### Lymphedema

Lymphedema after breast cancer treatment occurs in 14–40% of patients with localized breast cancer with the vast majority of cases occurring in the first 24 months after treatment but can arise many years later. [54] With modern sentinel lymph node sampling procedures, estimated breast-cancer-associated lymphedema risk is lowered to 6–10%. [55] Several factors are associated with increased risk for lymphedema including axillary lymph nodal dissection, radiation to the breast, axillary, internal mammary or sub clavicular lymph nodes, resection of a higher number of lymph nodes, capsular invasion by malignancy, postoperative complications, impairment of venous circulation due to thrombosis, indwelling port, a pacemaker or indwelling dialysis catheter, recurrent or advanced cancer, and obesity. [56] Lymphedema may manifest as arm or hand heaviness or discomfort and this is generally a clinical diagnosis characterized by disproportionate size of the affected arm. [57] Generally, it is recommended to avoid venipuncture, injections, and obtaining blood pressure on the side of the body that was operated upon. However, recent data from prospective and observational studies do not consistently validate this. [58] Treatment comprises physiotherapeutic interventions like decongestive lymphatic treatment. This entails skin care, exercise, and bandaging in multiple layers. Many observational studies have demonstrated that microsurgical procedures such as lymphovenous anastomosis lead to a decrease in limb circumference or volume following this surgery. [59]The lymphovenous bypass is a microsurgical approach performed at the time of axillary lymph node dissection with a pre-emptive intent to minimize the risk of lymphedema. [60] A recent preliminary analysis from a randomized control study from Memorial Sloan Kettering is showing promising data favoring immediate lymphatic reconstruction following axillary lymph node dissection decreasing the incidence of breast cancer-related lymphedema. [61] In addition, surgical liposuction has also been shown to ameliorate fatty fibrosis which may be seen with lymphedema. [62] Most academic guidelines such as The National Lymphedema Network, the International Society of Lymphology, the National Accreditation Program for Breast Centers, and the National Comprehensive Cancer Network make best practice recommendations regarding preoperative evaluation and subsequent surveillance of the ipsilateral and contralateral arms at regular standardized time points. [63, 66].

#### Delayed Adverse Effects of Immunotherapy

Delayed side effects of immune checkpoint inhibitors (ICI) in breast cancer survivors can occur months or even years after completing treatment. Endocrine disorders, including hypo or hyperthyroidism, adrenal insufficiency, hypophysitis, and type I diabetes, affect approximately 10% of ICI - treated patients [67]. These conditions often require lifelong hormonal supplementation and regular endocrine follow-up. Additionally, endocrine dysfunction may also have a delayed onset, occurring between 14 and 52 weeks after ICI treatment, as shown in a study based on breast cancer-specific cohort, emphasizing the need for extended monitoring [68].

Dermatological toxicities, such as vitiligo, alopecia areata, and nail dystrophy, may appear as late onset adverse events. These conditions often persist even after ICI discontinuation and are typically managed with high-potency steroids [69]. Bullous pemphigoid, with an onset ranging from 1 to 17 months post-treatment, can also develop after cessation of ICIs, requiring tailored management [70]. Other rare but significant skin-related issues include keratoacanthoma and cutaneous squamous cell carcinoma, as reported in the US Food and Drug Administration's (FDA) Adverse Event Reporting System (2004–2023). These are generally addressed with conservative measures like steroids, intralesional chemotherapy, cryotherapy or surgical excision [71].

Pulmonary complications, such as pneumonitis, represent a more serious delayed side effect, with cases reported up to 19 months after ICI initiation. Symptoms often include cough, worsening hypoxia, and radiographic findings consistent with organizing pneumonia or nonspecific interstitial pneumonitis [72, 73]. Management typically involves systemic glucocorticoids, with immunosuppressive therapy required in steroid-refractory cases but some cases may be irreversible.

Although uncommon, patients may develop neurological or cardiac complications that significantly affect long-term survival and quality of life. Conditions such as encephalitis and myasthenia gravis, though rare, can be life-threatening, while issues like peripheral neuropathy, myositis, and chronic headaches may lead to persistent, long-term medical challenges [74]. Similarly, myocarditis, while potentially fatal, can also result in lasting complications such as congestive heart failure or cardiac arrhythmias [75].

Other less common delayed effects include ocular toxicities (dry eye, episcleritis, or uveitis), hematologic conditions (immune thrombocytopenia, neutropenia, or aplastic anemia), and renal complications (interstitial nephritis, minimal change disease, or worsening hypertension). Although these effects are rare, they may significantly impact long-term outcomes and require careful management [74].

Given the range and severity of these delayed adverse effects, long-term follow-up with a multidisciplinary care team is essential to ensure timely identification and management, improving both survival and quality of life for breast cancer survivors.

#### Musculoskeletal Symptoms

Musculoskeletal symptoms such as joint pain, stiffness, myalgia, and bone pain are among the most common side effects experienced by women taking aromatase inhibitors (AI) [76]. These symptoms, collectively referred to as aromatase inhibitor-associated musculoskeletal syndrome (AIMSS), affect an estimated 20–50% of patients and can significantly impact adherence to AI therapy. Poor compliance and early discontinuation of AI due to these side effects have been widely reported and can adversely affect overall survival rates [77, 78].

Nonsteroidal anti-inflammatory drugs (NSAIDs) remain the primary pharmacological option for managing AIMSS due to their proven efficacy in managing inflammatory pain and their favorable safety profile. For patients who experience limited relief from NSAIDs, duloxetine offers an alternative option. It was shown to improve joint pain over a 12-week treatment period and was generally well tolerated, though its effects were similar to placebo at the 24-week mark [79]. Another viable strategy is temporarily discontinuing AIs and transitioning to a different endocrine therapy. Prospective studies indicate that 60–70% of patients can tolerate an alternate AI for at least six months following this approach [80].

Complementary interventions such as Vitamin D, Vitamin E, omega-3 fatty acids, and glucosamine/chondroitin have been evaluated in RCTs and cohort studies. However, none of these supplements demonstrated significant benefits in managing AIMSS [81].

A comprehensive strategy for managing AIMSS includes combining pharmacological treatments with physical exercise. In the randomized HOPE study, an exercise regimen significantly reduced worst pain scores and pain severity compared to usual care, with greater improvements observed among patients who attended more sessions [82]. While similar results have not been consistently observed in RCTs evaluating interventions like Nordic walking or mixed aerobic/resistance exercises, findings from the HOPE study, along with several non-randomized studies, highlight the broader health benefits of regular exercise, making it a highly recommended option for patients [81].

Acupuncture represents another nonpharmacological option for patients with AIMSS. A meta-analysis of two RCTs showed a non-significant decrease in worst pain scores after acupuncture [81]. However, a larger RCT comparing true acupuncture to sham acupuncture or a waitlist control group found a statistically significant reduction in joint pain at six weeks. While the clinical significance of this small improvement remains uncertain, acupuncture may still be considered for patients seeking safer alternative therapies [83].

#### Neuropathy

Peripheral neuropathy (PN) is estimated to affect 11–80% of survivors of early-stage breast cancer. [84] In the LILAC study of post-menopausal breast cancer survivors, 17% reported peripheral neuropathy (PN), with about 75% experiencing persistent symptoms for an average of 6.5 years. Variation in PN prevalence across studies likely stems from differences in populations, treatments, assessment timing, and reporting methods. [85].

Chemotherapy-induced peripheral neuropathy (CIPN) is a common, debilitating long-term side effect that impairs function and quality of life in breast cancer survivors, often caused by taxanes, platinum compounds, vinca alkaloids, eribulin mesylate and ixabepilone [86, 88]. Antibody–drug conjugates (ADCs) like trastuzumab emtansine (T-DM1), trastuzumab deruxtecan, and sacituzumab govitecan can also cause PN. T-DM1 carries the highest risk due to its microtubule inhibitor payload, while the other two ADCs have topoisomerase inhibitor payloads, which pose a lower risk. The mechanism of PN in these patients is similar to CIPN, allowing for similar strategies for risk assessment, prevention, and treatment [89].

CIPN typically causes predominantly sensory symptoms like numbness, pain, and hypersensitivity in the hands and feet, often resembling a"glove and stocking"pattern and leads to difficulty with daily tasks, such as fine motor skills and walking. CIPN may worsen even after chemotherapy ends, in a phenomenon called"coasting."[90, 91] CIPN in breast cancer patients often causes chemotherapy dose reductions or discontinuation, with persistent sensory and motor symptoms impairing quality of life, function, and finances in survivors [92, 93].

Higher BMI, black race, genetic predisposition, pre-chemotherapy PN, younger age at diagnosis, post-menopausal status, renal dysfunction with reduced creatinine clearance, and history of smoking have been identified as risk factors for developing CIPN [85, 94, 101]. Poorly controlled diabetes mellitus (DM) is associated with DM-induced sensory neuropathy but its role as an independent risk factor for developing CIPN is not clear. Paclitaxel is associated with greater risk of CIPN than docetaxel and it also can cause an acute pain syndrome in the days following its administration [94, 96, 102, 103].

The pathophysiology of chemotherapy-induced peripheral neuropathy (CIPN) are multifactorial, involving microtubule disruption, oxidative stress, mitochondrial damage, ion channel changes, myelin damage, DNA damage, and neuroinflammation. Novel study models with molecular approach show promise in identifying potential therapeutic targets for prevention and treatment of CIPN [101, 104, 111, 112].

The most common preventive intervention in clinical setting for the breast cancer patients remains CIPN-associated dose reduction of chemotherapy [92] In the NSABP B-30 trial, cumulative taxane dose was strongly associated with higher rates of long-term chemotherapy-induced PN [113] Given the modest differences in overall and disease-free survival between various taxane-containing regimens, clinicians should collaborate with breast cancer patients to make informed decisions about selecting and dosing adjuvant taxane therapies. This decision should consider the patient's risk factors for developing CIPN [113, 114].

Despite the advancements in pre-clinical research and ongoing clinical research on the prevention of CIPN, the updated 2020 ASCO guidelines did not recommend specific interventions to prevent CIPN outside of a clinical trial while acknowledging preliminary evidence for potential benefit from acupuncture, cryotherapy, compression therapy, exercise therapy, and ganglioside-monosialic acid (GM-1) [115] Recently published clinical trials and a meta-analysis of 442 patients enrolled in 4 randomized clinical trials (RCTs) provides supporting evidence for the clinical benefit from compression therapy in preventing CIPN [116, 119]. Cumulative evidence from clinical studies also continues to demonstrate a preventive effect of cryotherapy or “cooling of hands and feet* against the development of CIPN [120, 122]. The Cropsi study randomized 200 patients 1:1 between cryotherapy and cryocompression interventions and both were reported as safe and effective for prevention of CIPN [123]. More definitive answers will likely come from an NCI approved cooperative group phase III clinical trial conducted by ALLIANCE/SWOG that will randomize patients receiving a taxane to one of three arms: compression therapy, combined compression and cryotherapy, or a control group (the STOP CIPN trial, SWOG 2205). Enrollment is ongoing. Acupuncture and electroacupuncture have also demonstrated potential clinical benefits in the prevention of CIPN; however, larger randomized, prospective studies are necessary to confirm their efficacy [124, 125]. A recently published RCT of 47 patients has demonstrated effectiveness of duloxetine in preventing paclitaxel induced PN [126]. Ongoing clinical trials are studying the use of surgical gloves and photobiomodulation in prevention of CIPN [127, 128].

For the patients who have already developed CIPN and have completed neurotoxic chemotherapy, the updated 2020 ASCO guidelines recommended the clinicians to offer duloxetine as the only pharmacologic agent with consistent evidence supporting its efficacy in reducing CIPN pain [129, 131]. No final recommendations were made for using exercise therapy, acupuncture, gabapentinoids, TCAs, or cannabinoids outside of a clinical trial [115].

Exercise, including exercise programs and resistance training has shown beneficial outcomes in reducing severity of CIPN and improving subjective and objective assessments of CIPN and quality of life for cancer survivors [132, 136]. Acupuncture and Electroacupuncture have both shown utility and benefit in ameliorating symptoms of CIPN in RCTs [137, 138]. Yoga is another feasible and non-pharmacological modality for treatment of CIPN that has a potential to improve functionality and symptoms burden of CIPN in breast cancer survivors [139]. While there are ongoing Phase III RCTs for evaluation of acupuncture and yoga for CIPN, they should be considered for patients with CIPN given lack of other safe and effective options for these patients [140, 141]. Intermittent hand-foot hypothermia therapy might be useful in improving CIPN symptoms [142]. The evidence for using gabapentinoids in CIPN is mixed but a recent study evaluating mirogabalin showed effectiveness for patients with moderate to severe CIPN [143, 145]. Novel approaches are focusing on neurofeedback, topical modulation of epidermal nociceptor receptors, and utilizing pharmacological agents with pre-clinical activity to treat symptoms of CIPN [146, 148].

It is important to note that inconsistent outcome measures, heterogenous control group selection, and variable exposure duration have hindered the interpretation of several studies for prevention and treatment of CIPN in the past. Ongoing NCI-sponsored research aims to better define the CIPN phenotype and standardize outcome measures for future studies. There is no universally agreed upon method to assess CIPN, but patient reported outcomes as measured by standardized questionnaires including EORTC-CIPN20, FACT/GOG-Ntx and PRO-CTCAE have shown to be most optimal for capturing severity and responsiveness of CIPN [149].

Ongoing clinical trials exploring novel pharmacological and neuro-interventional approaches, along with the integration of pharmacogenetics and multi-omics into CIPN risk-assessment models, promise improved strategies for the prevention and management of CIPN in breast cancer survivors.

#### Non-CIPN Neuropathy

Immune checkpoint inhibitor (ICI)-associated PN involves a wide spectrum of clinical manifestations with involvement of sensory, motor and/or autonomic nerves. Cranial neuropathies and inflammatory polyradiculopathies, such as Guillain Barre syndrome, are the most frequently reported. Risk factors remain unclear, and diagnosis and management differs from CIPN due to its immune mediated etiology. [150, 151]. The novel targeted agents in breast cancer like CDK4/6 inhibitors (e.g., palbociclib) and PARP inhibitors (e.g., rucaparib, olaparib) have been linked to mild neurotoxicities that manifest as dysgeusia and myalgias [88, 152].

Radiation-induced brachial plexopathy (RIBP) is a late-onset, disabling neuropathic complication of adjuvant radiotherapy, commonly observed in breast cancer survivors. Currently, no validated therapeutic strategies exist for RIBP, with rehabilitation primarily involving physical, occupational, and hand therapies [153, 154]. Finally, postmastectomy pain syndrome (PMPS) is a chronic, debilitating form of persistent postsurgical pain with neuropathic features. Patients often experience burning, stabbing, or pulling sensations in the anterior thorax, axilla, or upper medial arm after mastectomy, lumpectomy, or similar surgeries. Treatment options include pharmacological therapies (e.g., TCAs, SNRIs) and interventional approaches such as peripheral nerve surgery, peripheral nerve blocks, radiofrequency ablation, or botulinum injections [155, 157].

## Role of ctDNA in Breast Cancer

The growing use of circulating tumor DNA (ctDNA) in solid tumors has introduced a noninvasive way to detect minimal residual disease (MRD), which refers to the presence of molecular evidence of cancer in the absence of overt clinical or radiographic metastasis or recurrence [158]. Emerging evidence suggests ctDNA can help predict treatment response and the likelihood of metastatic relapses across multiple cancer types [159, 161]. Studies also indicate that detecting ctDNA after neoadjuvant therapy correlates with a higher risk of recurrence, regardless of residual disease status [162, 163]. A recent study of 78 patients with nonmetastatic breast cancer used whole genome sequencing to create personalized ctDNA panels. Among 63 patients who remained recurrence-free, 60 showed ctDNA clearance, whereas all 11 patients who relapsed had detectable MRD. These findings suggest that post-operative ctDNA clearance is strongly linked to better recurrence-free and cancer-specific survival, supporting its potential for early relapse detection [164]. However, most of the data in ctDNA realm come from retrospective analyses of prospectively collected samples, leaving unanswered whether early MRD detection via ctDNA actually improves outcomes. Prospective trials, like the ongoing SURVIVE study (NCT05658172), aim to clarify this by comparing standard surveillance with intensive monitoring, including tumor-informed ctDNA analysis, in early breast cancer patients after curative treatment [165].

While ctDNA testing is not yet routinely recommended in major guidelines, three commercial tests are currently available in the U.S.: two tumor-informed assays (Signatera™ and RaDaR™) and one tumor-agnostic test (Guardant Reveal™). As novel research and clinical trials are conducted, ctDNA may become a valuable tool for guiding treatment decisions and improving long-term outcomes. [166].

### Bone Mineral Density in Breast Cancer Survivors

Breast cancer treatments such as gonadotropin-releasing hormone (GnRH) analogs, chemotherapy, and aromatase inhibitors (AIs) can contribute to bone loss and increase fracture risk. Clinical trials have shown that women taking anastrozole or exemestane experience greater bone mineral density (BMD) loss compared to those on tamoxifen [167, 168]. Meta-analyses of adjuvant trials further confirm that AIs significantly increase fracture risk relative to tamoxifen or placebo, with extended endocrine therapy duration correlating with higher risk [169, 170]. Given these risks, it is essential to assess BMD via dual-energy x-ray absorptiometry (DEXA) scans at baseline and at regular intervals for women on long-term endocrine therapy [168]. While there are no formal guidelines on DEXA scan frequency, monitoring should be individualized to each patient based on baseline T-scores and additional osteoporosis risk factors. Bone targeted therapies can be instituted in women with BMD T-score ≤ −2.5 or between −1.0 and −2.5 with additional risk factors for fracture, or history of fragility fractures [61, 167]. Bone-targeted therapies, such as IV or oral bisphosphonates, are typically first-line due to their cost-effectiveness and established safety profile. However, denosumab is a suitable alternative for patients who cannot tolerate bisphosphonates or have contraindications. Potential side effects of bisphosphonates include flu-like symptoms, renal impairment, atypical femur fractures, and osteonecrosis of the jaw. Notably, denosumab carries a risk of rebound fractures after discontinuation. The optimal duration of bone-protective therapy remains uncertain but should be tailored based on BMD trends, treatment response, and ongoing fracture risk.

### Fear of Cancer Recurrence

Fear of cancer recurrence is a common concern among breast cancer survivors, leading to emotional distress, anxiety, and depression. This psychological burden is often neglected, with many survivors seeking greater access to support [171, 174]. Breast cancer survivors often need detailed information about their condition, treatment side effects, and long-term care. Although the American Cancer Society and ASCO recommend comprehensive survivorship care plans, their implementation and perceived effectiveness remain inconsistent [175]. Unmet psychosocial needs remain widespread, with a Dutch survey reporting 94% of cancer survivors seeking support, particularly for fear of recurrence and access to psychological care [172]. Social support has been shown to play a critical role in alleviating fear of recurrence and enhancing survivors’ quality of life [171, 172, 176] There is significant demand for peer support programs, allowing survivors to share experiences and receive emotional support. One-on-one peer support has proven effective in meeting many of these needs [177]. Survivors often require help with daily tasks, including managing household duties and navigating healthcare systems [178]. Sexual health and intimacy issues are common among survivors, yet they often report insufficient support and information in this area [173, 179] Effective coordination of care, including follow-up and comorbidity management, is vital; however, many survivors experience gaps, particularly during the transition from active treatment to survivorship [180]. Healthcare providers must address fear of recurrence and ensure access to psychological support in survivorship care. A comprehensive approach, including standardized assessments and targeted interventions, is essential to improve breast cancer survivors'quality of life.

### Role of GLP-1 agonists in Breast Cancer

Weight loss in breast cancer survivors is linked to reduced recurrence risk and improved prognosis. RCTs show dietary changes supporting weight loss can enhance outcomes. ASCO guidelines endorse weight management, diet, and exercise to improve quality of life and mitigate treatment related side effects [175, 181] GLP-1 receptor agonists, including semaglutide and liraglutide, have demonstrated clinically meaningful weight loss in breast cancer patients, with studies reporting a 5% mean weight reduction at 12 months and up to 8.89 kg in women with stage I–III disease [182, 183]. These agents are endorsed by the AGA for obesity management in adults [184] Emerging preclinical data also suggest potential antineoplastic effects, with GLP-1 analogs shown to activate AMPK and inhibit NF-κB, mechanisms known to suppress tumor growth and proliferation [185, 186]. Additionally, a large population-based study found an association between GLP-1 agonist use and reduced breast cancer risk in obese individuals [187]. Despite promising findings, prospective clinical studies are needed to clarify the long-term effects of GLP-1 agonists on breast cancer recurrence, progression, and survivorship [182, 183].

### Lifestyle Interventions (diet, exercise) to Improve the Outcomes

Multiple studies have demonstrated that obesity is a poor prognostic factor for breast cancer. Weight loss interventions such as telephone-based interventions in the Breast Cancer Weight Loss (BWEL study), motivational interviewing and text interventions to optimize adherence to endocrine therapy for breast cancer (GETSET Alliance trial) demonstrate mitigation strategies to optimize outcomes. There are also multiple studies evaluating the roles of different diets and fasting or time-restricted eating pattern, gut microbiome modulation, and their impact on cancer outcomes [82, 188, 190].

### Anthracycline-related Cardiotoxicity

Anthracyclines such as doxorubicin, epirubicin are one of the most potent therapeutic options for breast cancer patients across the different breast cancer histologies. However, this is associated with significant acute and chronic cardiotoxicity risk. This can include heart failure, coronary artery disease. Most toxicities are dose dependent and cumulative. The recommended monitoring guidelines recommend clinical assessment in conjunction with transthoracic echocardiography and cardiac biomarkers (natriuretic peptides, cardiac troponin). Heart failure risk was higher for patients with cancer receiving anthracycline (HR, 3.25 [95% CI, 2.11–5.00]; *P* < 0.001 and greater total incidence for patients treated with anthracyclines vs comparator group was noted at 1 year (1.81% vs 0.09%), 5 years (2.91% vs 0.79%), 10 years (5.36% vs 1.74%) (*P* < 0.001). In recent years, the balancing act between risk vs benefit for the addition of anthracyclines has led to their usage being reserved for higher risk cancers like estrogen driven cancers (tumors with multiple nodes, higher risk genomic scores including oncotype score over 30 or mammaprint high risk 2), triple negative breast cancer and less commonly for HER2 driven cancers [191] The recent SCARLET trial is currently evaluating potential omission of anthracyclines for neoadjuvant treatment of triple negative breast cancer [192, 195]. Preventive strategies involving cardio-oncologists and utilizing drugs such as beta blockers, aldosterone antagonists, angiotensin converting enzyme inhibitors, statins and dexrazoxane have been found to be helpful.

### Cognitive Impairment

As the armamentarium of cancer treatment broadens, we are beginning to notice profound and long-term effects on cognitive function. Cancer-associated cognitive decline encompasses issues relating to focus, memory, attention, executive functioning and psychomotor speed). While for many patients this may be transient, with improvements being noted slowly over a couple of years; however for 20–30% of patients, the recovery may be incomplete and may linger on for many years [196] These may be noted with systemic therapy options, including chemotherapy, endocrine therapy, immunotherapy, and other targeted agents used on breast cancer. Various therapies such as donepezil, memantine, gingko biloba, CNS stimulants including modafinil and methylphenidate, have been evaluated to mitigate the cognitive decline. In addition, physical exercise and meditation have also been considered helpful approaches [197, 199] Ongoing studies such as MIND-BC and MIND-TNBC are aiming to address this issue with Mediterranean-Dietary Approaches to Stop Hypertension Intervention for Neurodegenerative Delay (MIND), on mental (cognitive) changes in women with triple negative breast cancer (TNBC) undergoing active treatment.

### Secondary Malignancies Associated with Breast Cancer Treatment

Patients with breast cancer are at a higher risk of developing myelodysplastic syndrome or leukemia related to systemic chemotherapy, endometrial cancer related to tamoxifen use, and angiosarcoma related to radiation. Routine surveillance by oncologists or general physicians should include monitoring for these secondary malignancies with clinical encounters [200, 201]

## Conclusion

Comprehensive breast cancer survivorship care is complex and includes detection of recurrence/new cancers, monitoring and managing short and long term side effects of treatment, and management of chronic conditions.

## Key References

- Partridge AH, Niman SM, Ruggeri M, Peccatori FA, Azim HA, Colleoni M, et al. Interrupting Endocrine Therapy to Attempt Pregnancy after Breast Cancer. New England Journal of Medicine. 2023;388(18):1645-56
  - This study evaluates the safety and feasibility of temporarily interrupting endocrine therapy to attempt pregnancy in breast cancer survivors
- Lederman S, Ottery FD, Cano A, Santoro N, Shapiro M, Stute P, et al. Fezolinetant for treatment of moderate-to-severe vasomotor symptoms associated with menopause (SKYLIGHT 1): a phase 3 randomised controlled study. Lancet. 2023;401(10382):1091- 102.
  - In this phase 3 randomized controlled trial demonstrates the efficacy of fezolinetant in reducing moderate-to-severe vasomotor symptoms associated with menopause.
- McVicker L, Labeit AM, Coupland CAC, Hicks B, Hughes C, McMenamin Ú, et al. Vaginal Estrogen Therapy Use and Survival in Females With Breast Cancer. JAMA Oncol. 2024;10(1):103-8.
  - The study investigates the impact of vaginal estrogen therapy on survival outcomes in females with breast cancer indicating situations in which this may be an efficacious treatment option.
- Lambertini M, Arecco L, Woodard TL, Messelt A, Rojas KE. Advances in the Management of Menopausal Symptoms, Fertility Preservation, and Bone Health for Women With Breast Cancer on Endocrine Therapy. American Society of Clinical Oncology Educational Book. 2023(43):e390442.
  - This review highlights advances in managing menopausal symptoms, fertility preservation, and bone health in women undergoing endocrine therapy for breast cancer.
- Coriddi M, Dayan J, Bloomfield E, McGrath L, Diwan R, Monge J, et al. Efficacy of Immediate Lymphatic Reconstruction to Decrease Incidence of Breast Cancer-related Lymphedema: Preliminary Results of Randomized Controlled Trial. Ann Surg. 2023;278(4):630-7
  - Preliminary randomized controlled data from this trial suggests that immediate lymphatic reconstruction may reduce the incidence of breast cancer-related lymphedema.

## Data Availability

No datasets were generated or analysed during the current study.

## References

[1] Harbeck N, Gnant M. Breast cancer. Lancet. 2017;389(10074):1134–50. 27865536 10.1016/S0140-6736(16)31891-8

[2] Sopik V. International variation in breast cancer incidence and mortality in young women. Breast Cancer Res Treat. 2021;186(2):497–507. 33145697 10.1007/s10549-020-06003-8

[3] Ellington TD, Miller JW, Henley SJ, Wilson RJ, Wu M, Richardson LC. Trends in Breast Cancer Incidence, by Race, Ethnicity, and Age Among Women Aged ≥20 Years - United States, 1999–2018. MMWR Morb Mortal Wkly Rep. 2022;71(2):43–7. 35025856 10.15585/mmwr.mm7102a2 PMC8757618

[4] Jordan K, Aapro M, Kaasa S, Ripamonti CI, Scotté F, Strasser F, et al. European Society for Medical Oncology (ESMO) position paper on supportive and palliative care. Ann Oncol. 2018;29(1):36–43. 29253069 10.1093/annonc/mdx757

[5] Siegel RL, Giaquinto AN, Jemal A. Cancer statistics, 2024. CA Cancer J Clin. 2024;74(1):12–49. 38230766 10.3322/caac.21820

[6] Soldato D, Arecco L, Agostinetto E, Franzoi MA, Mariamidze E, Begijanashvili S, et al. The Future of Breast Cancer Research in the Survivorship Field. Oncol Ther. 2023;11(2):199–229. 37005952 10.1007/s40487-023-00225-8 PMC10260743

[7] Ruggeri M, Pagan E, Bagnardi V, Bianco N, Gallerani E, Buser K, et al. Fertility concerns, preservation strategies and quality of life in young women with breast cancer: Baseline results from an ongoing prospective cohort study in selected European Centers. Breast. 2019;47:85–92. 31362134 10.1016/j.breast.2019.07.001

[8] Ruddy KJ, Gelber SI, Tamimi RM, Ginsburg ES, Schapira L, Come SE, et al. Prospective study of fertility concerns and preservation strategies in young women with breast cancer. J Clin Oncol. 2014;32(11):1151–6. 24567428 10.1200/JCO.2013.52.8877 PMC4164759

[9] Sella T, Poorvu PD, Ruddy KJ, Gelber SI, Tamimi RM, Peppercorn JM, et al. Impact of fertility concerns on endocrine therapy decisions in young breast cancer survivors. Cancer. 2021;127(16):2888–94. 33886123 10.1002/cncr.33596 PMC8351455

[10] Martelli V, Latocca MM, Ruelle T, Perachino M, Arecco L, Beshiri K, et al. Comparing the Gonadotoxicity of Multiple Breast Cancer Regimens: Important Understanding for Managing Breast Cancer in Pre-Menopausal Women. Breast Cancer (Dove Med Press). 2021;13:341–51. 34079366 10.2147/BCTT.S274283 PMC8164347

[11] Lambertini M, Peccatori FA, Demeestere I, Amant F, Wyns C, Stukenborg JB, et al. Fertility preservation and post-treatment pregnancies in post-pubertal cancer patients: ESMO Clinical Practice Guidelines(†). Ann Oncol. 2020;31(12):1664–78. 32976936 10.1016/j.annonc.2020.09.006

[12] Anderson RA, Amant F, Braat D, D'Angelo A, Chuva de Sousa Lopes SM, Demeestere I, et al ESHRE guideline: female fertility preservation. Hum Reprod Open. 2020;2020(4):hoaa052. 10.1093/hropen/hoaa052 PMC7666361 33225079

[13] Letourneau JM, Ebbel EE, Katz PP, Katz A, Ai WZ, Chien AJ, et al. Pretreatment fertility counseling and fertility preservation improve quality of life in reproductive age women with cancer. Cancer. 2012;118(6):1710–7. 21887678 10.1002/cncr.26459 PMC3235264

[14] Lambertini M, Horicks F, Del Mastro L, Partridge AH, Demeestere I. Ovarian protection with gonadotropin-releasing hormone agonists during chemotherapy in cancer patients: From biological evidence to clinical application. Cancer Treat Rev. 2019;72:65–77. 30530271 10.1016/j.ctrv.2018.11.006

[15] Lambertini M, Moore HCF, Leonard RCF, Loibl S, Munster P, Bruzzone M, et al. Gonadotropin-Releasing Hormone Agonists During Chemotherapy for Preservation of Ovarian Function and Fertility in Premenopausal Patients With Early Breast Cancer: A Systematic Review and Meta-Analysis of Individual Patient-Level Data. J Clin Oncol. 2018;36(19):1981–90. 29718793 10.1200/JCO.2018.78.0858 PMC6804855

[16] Lambertini M, Blondeaux E, Bruzzone M, Perachino M, Anderson RA, de Azambuja E, et al. Pregnancy After Breast Cancer: A Systematic Review and Meta-Analysis. J Clin Oncol. 2021;39(29):3293–305. 34197218 10.1200/JCO.21.00535

[17] Lambertini M, Di Maio M, Pagani O, Curigliano G, Poggio F, Del Mastro L, et al. The BCY3/BCC 2017 survey on physicians’ knowledge, attitudes and practice towards fertility and pregnancy-related issues in young breast cancer patients. Breast. 2018;42:41–9. 30170202 10.1016/j.breast.2018.08.099

[18] Anderson RA, Lambertini M, Hall PS, Wallace WH, Morrison DS, Kelsey TW. Survival after breast cancer in women with a subsequent live birth: Influence of age at diagnosis and interval to subsequent pregnancy. Eur J Cancer. 2022;173:113–22. 35868140 10.1016/j.ejca.2022.06.048

[19] Burstein HJ, Lacchetti C, Anderson H, Buchholz TA, Davidson NE, Gelmon KE, et al. Adjuvant Endocrine Therapy for Women With Hormone Receptor-Positive Breast Cancer: American Society of Clinical Oncology Clinical Practice Guideline Update on Ovarian Suppression. J Clin Oncol. 2016;34(14):1689–701. 26884586 10.1200/JCO.2015.65.9573

[20] Lambertini M, Kroman N, Ameye L, Cordoba O, Pinto A, Benedetti G, et al. Long-term Safety of Pregnancy Following Breast Cancer According to Estrogen Receptor Status. J Natl Cancer Inst. 2018;110(4):426–9. 29087485 10.1093/jnci/djx206 PMC6658852

[21] Partridge AH, Niman SM, Ruggeri M, Peccatori FA, Azim HA Jr, Colleoni M, et al. Who are the women who enrolled in the POSITIVE trial: A global study to support young hormone receptor positive breast cancer survivors desiring pregnancy. Breast. 2021;59:327–38. 34390999 10.1016/j.breast.2021.07.021 PMC8365381

[22] Partridge AH, Niman SM, Ruggeri M, Peccatori FA, Azim HA, Colleoni M, et al. Interrupting Endocrine Therapy to Attempt Pregnancy after Breast Cancer. N Engl J Med. 2023;388(18):1645–56. 37133584 10.1056/NEJMoa2212856 PMC10358451

[23] Duffy C, Allen S. Medical and psychosocial aspects of fertility after cancer. Cancer J. 2009;15(1):27–33. 19197170 10.1097/PPO.0b013e3181976602 PMC2719717

[24] Sessa C, Balmaña J, Bober SL, Cardoso MJ, Colombo N, Curigliano G, et al. Risk reduction and screening of cancer in hereditary breast-ovarian cancer syndromes: ESMO Clinical Practice Guideline. Ann Oncol. 2023;34(1):33–47. 36307055 10.1016/j.annonc.2022.10.004

[25] Blondeaux E, et al. Association between risk-reducing surgeries and survival in young BRCA carriers with breast cancer: an international cohort study. Lancet Oncol. 2025;26(6):759–70. 10.1016/S1470-2045(25)00152-4 40347973

[26] Turan V, Lambertini M, Lee DY, Wang E, Clatot F, Karlan BY, et al. Association of Germline BRCA Pathogenic Variants With Diminished Ovarian Reserve: A Meta-Analysis of Individual Patient-Level Data. J Clin Oncol. 2021;39(18):2016–24. 33891474 10.1200/JCO.20.02880 PMC8260903

[27] Valentini A, Finch A, Lubinski J, Byrski T, Ghadirian P, Kim-Sing C, et al. Chemotherapy-induced amenorrhea in patients with breast cancer with a BRCA1 or BRCA2 mutation. J Clin Oncol. 2013;31(31):3914–9. 23980083 10.1200/JCO.2012.47.7893 PMC3805929

[28] Thompson JA. New NCCN Guidelines: Recognition and Management of Immunotherapy-Related Toxicity. J Natl Compr Canc Netw. 2018;16(5s):594–6. 29784734 10.6004/jnccn.2018.0047

[29] Franzoi MA, Agostinetto E, Perachino M, Del Mastro L, de Azambuja E, Vaz-Luis I, et al. Evidence-based approaches for the management of side-effects of adjuvant endocrine therapy in patients with breast cancer. Lancet Oncol. 2021;22(7):e303–13. 33891888 10.1016/S1470-2045(20)30666-5

[30] Mann E, Smith MJ, Hellier J, Balabanovic JA, Hamed H, Grunfeld EA, et al. Cognitive behavioural treatment for women who have menopausal symptoms after breast cancer treatment (MENOS 1): a randomised controlled trial. Lancet Oncol. 2012;13(3):309–18. 22340966 10.1016/S1470-2045(11)70364-3 PMC3314999

[31] Pandya KJ, Morrow GR, Roscoe JA, Zhao H, Hickok JT, Pajon E, et al. Gabapentin for hot flashes in 420 women with breast cancer: a randomised double-blind placebo-controlled trial. Lancet. 2005;366(9488):818–24. 16139656 10.1016/S0140-6736(05)67215-7 PMC1627210

[32] Jin Y, Desta Z, Stearns V, Ward B, Ho H, Lee KH, et al. CYP2D6 genotype, antidepressant use, and tamoxifen metabolism during adjuvant breast cancer treatment. J Natl Cancer Inst. 2005;97(1):30–9. 15632378 10.1093/jnci/dji005

[33] Loprinzi CL, Kugler JW, Sloan JA, Mailliard JA, LaVasseur BI, Barton DL, et al. Venlafaxine in management of hot flashes in survivors of breast cancer: a randomised controlled trial. Lancet. 2000;356(9247):2059–63. 11145492 10.1016/S0140-6736(00)03403-6

[34] Loprinzi CL, Sloan JA, Perez EA, Quella SK, Stella PJ, Mailliard JA, et al. Phase III evaluation of fluoxetine for treatment of hot flashes. J Clin Oncol. 2002;20(6):1578–83. 11896107 10.1200/JCO.2002.20.6.1578

[35] Goldberg RM, Loprinzi CL, O’Fallon JR, Veeder MH, Miser AW, Mailliard JA, et al. Transdermal clonidine for ameliorating tamoxifen-induced hot flashes. J Clin Oncol. 1994;12(1):155–8. 8270972 10.1200/JCO.1994.12.1.155

[36] Leon-Ferre RA, Novotny PJ, Wolfe EG, Faubion SS, Ruddy KJ, Flora D, et al. Oxybutynin vs Placebo for Hot Flashes in Women With or Without Breast Cancer: A Randomized, Double-Blind Clinical Trial (ACCRU SC-1603). JNCI Cancer Spectr. 2020;4(1):pkz088. 32337497 10.1093/jncics/pkz088 PMC7050158

[37] Lederman S, Ottery FD, Cano A, Santoro N, Shapiro M, Stute P, et al. Fezolinetant for treatment of moderate-to-severe vasomotor symptoms associated with menopause (SKYLIGHT 1): a phase 3 randomised controlled study. Lancet. 2023;401(10382):1091–102. 36924778 10.1016/S0140-6736(23)00085-5

[38] Chen YP, Liu T, Peng YY, Wang YP, Chen H, Fan YF, et al. Acupuncture for hot flashes in women with breast cancer: A systematic review. J Cancer Res Ther. 2016;12(2):535–42. 27461606 10.4103/0973-1482.172716

[39] Condorelli M, Bruzzone M, Ceppi M, Ferrari A, Grinshpun A, Hamy AS, et al. Safety of assisted reproductive techniques in young women harboring germline pathogenic variants in BRCA1/2 with a pregnancy after prior history of breast cancer. ESMO Open. 2021;6(6): 100300. 34775302 10.1016/j.esmoop.2021.100300 PMC8593447

[40] Dos Santos CCM, Uggioni MLR, Colonetti T, Colonetti L, Grande AJ, Da Rosa MI. Hyaluronic Acid in Postmenopause Vaginal Atrophy: A Systematic Review. J Sex Med. 2021;18(1):156–66. 33293236 10.1016/j.jsxm.2020.10.016

[41] Goetsch MF, Lim JY, Caughey AB. A Practical Solution for Dyspareunia in Breast Cancer Survivors: A Randomized Controlled Trial. J Clin Oncol. 2015;33(30):3394–400. 26215946 10.1200/JCO.2014.60.7366 PMC4979375

[42] Management of symptomatic vulvovaginal atrophy: 2013 position statement of The North American Menopause Society. Menopause. 2013;20(9):888–902; quiz 3–4. 10.1097/GME.0b013e3182a122c2 23985562

[43] Amies Oelschlager AM, Debiec K. Vaginal Dilator Therapy: A Guide for Providers for Assessing Readiness and Supporting Patients Through the Process Successfully. J Pediatr Adolesc Gynecol. 2019;32(4):354–8. 31091469 10.1016/j.jpag.2019.05.002

[44] Santen RJ, Mirkin S, Bernick B, Constantine GD. Systemic estradiol levels with low-dose vaginal estrogens. Menopause. 2020;27(3):361–70. 31794498 10.1097/GME.0000000000001463 PMC7050796

[45] Le Ray I, Dell’Aniello S, Bonnetain F, Azoulay L, Suissa S. Local estrogen therapy and risk of breast cancer recurrence among hormone-treated patients: a nested case-control study. Breast Cancer Res Treat. 2012;135(2):603–9. 22903687 10.1007/s10549-012-2198-y

[46] Cold S, Cold F, Jensen MB, Cronin-Fenton D, Christiansen P, Ejlertsen B. Systemic or Vaginal Hormone Therapy After Early Breast Cancer: A Danish Observational Cohort Study. J Natl Cancer Inst. 2022;114(10):1347–54. 35854422 10.1093/jnci/djac112 PMC9552278

[47] McVicker L, Labeit AM, Coupland CAC, Hicks B, Hughes C, McMenamin Ú, et al. Vaginal Estrogen Therapy Use and Survival in Females With Breast Cancer. JAMA Oncol. 2024;10(1):103–8. 37917089 10.1001/jamaoncol.2023.4508 PMC10623297

[48] Barton DL, Sloan JA, Shuster LT, Gill P, Griffin P, Flynn K, et al. Evaluating the efficacy of vaginal dehydroepiandosterone for vaginal symptoms in postmenopausal cancer survivors: NCCTG N10C1 (Alliance). Support Care Cancer. 2018;26(2):643–50. 28921241 10.1007/s00520-017-3878-2 PMC5754227

[49] Faubion SS, Larkin LC, Stuenkel CA, Bachmann GA, Chism LA, Kagan R, et al. Management of genitourinary syndrome of menopause in women with or at high risk for breast cancer: consensus recommendations from The North American Menopause Society and The International Society for the Study of Women’s Sexual Health. Menopause. 2018;25(6):596–608. 29762200 10.1097/GME.0000000000001121

[50] Becorpi A, Campisciano G, Zanotta N, Tredici Z, Guaschino S, Petraglia F, et al. Fractional CO(2) laser for genitourinary syndrome of menopause in breast cancer survivors: clinical, immunological, and microbiological aspects. Lasers Med Sci. 2018;33(5):1047–54. 29492713 10.1007/s10103-018-2471-3

[51] Li FG, Maheux-Lacroix S, Deans R, Nesbitt-Hawes E, Budden A, Nguyen K, et al. Effect of Fractional Carbon Dioxide Laser vs Sham Treatment on Symptom Severity in Women With Postmenopausal Vaginal Symptoms: A Randomized Clinical Trial. JAMA. 2021;326(14):1381–9. 34636862 10.1001/jama.2021.14892 PMC8511979

[52] Nicoli Serquiz NS, Ayane C A Sarmento, Natalie R Almeida, Maria Luísa Nobre, Kleyton S Medeiros, Antonio C Q Aquino, Juliana D A S Camargo, Heitor D Medeiros, Beatriz B Siqueira, Ronnier Oliveira, Nícolas C Conrado, Larissa P Moura, Raphael M Carvalho, Ana Katherine Gonçalves. Use of intravaginal physical energy for treating genitourinary syndrome of menopause in breast cancer survivors: a systematic review. SABCS Abstract Number: SESS-3559. 2024. https://sabcs.org/Portals/0/Documents/FINAL%20SABCS%2024%20%20Poster%20Session%201%20.pdf?ver=COtfEZSxfP5lWkHrNkFAqg%3D%3D.

[53] Lambertini M, Arecco L, Woodard TL, Messelt A, Rojas KE. Advances in the Management of Menopausal Symptoms, Fertility Preservation, and Bone Health for Women With Breast Cancer on Endocrine Therapy. Am Soc Clin Oncol Educ Book. 2023;43: e390442. 37229618 10.1200/EDBK_390442

[54] Rockson SG, Rivera KK. Estimating the population burden of lymphedema. Ann N Y Acad Sci. 2008;1131:147–54. 18519968 10.1196/annals.1413.014

[55] Hayes S, Cornish B, Newman B. Comparison of methods to diagnose lymphoedema among breast cancer survivors: 6-month follow-up. Breast Cancer Res Treat. 2005;89(3):221–6. 15754119 10.1007/s10549-004-2045-x

[56] Iyigun ZE, Duymaz T, Ilgun AS, Alco G, Ordu C, Sarsenov D, et al. Preoperative Lymphedema-Related Risk Factors in Early-Stage Breast Cancer. Lymphat Res Biol. 2018;16(1):28–35. 28346852 10.1089/lrb.2016.0045

[57] Fu MR, Rosedale M. Breast cancer survivors’ experiences of lymphedema-related symptoms. J Pain Symptom Manage. 2009;38(6):849–59. 19819668 10.1016/j.jpainsymman.2009.04.030 PMC2795115

[58] Ferguson CM, Swaroop MN, Horick N, Skolny MN, Miller CL, Jammallo LS, et al. Impact of Ipsilateral Blood Draws, Injections, Blood Pressure Measurements, and Air Travel on the Risk of Lymphedema for Patients Treated for Breast Cancer. J Clin Oncol. 2016;34(7):691–8. 26644530 10.1200/JCO.2015.61.5948 PMC4872021

[59] Cornelissen AJM, Beugels J, Ewalds L, Heuts EM, Keuter XHA, Piatkowski A, et al. Effect of Lymphaticovenous Anastomosis in Breast Cancer-Related Lymphedema: A Review of the Literature. Lymphat Res Biol. 2018;16(5):426–34. 29356596 10.1089/lrb.2017.0067

[60] Boccardo F, Casabona F, De Cian F, Friedman D, Murelli F, Puglisi M, et al. Lymphatic microsurgical preventing healing approach (LYMPHA) for primary surgical prevention of breast cancer-related lymphedema: over 4 years follow-up. Microsurgery. 2014;34(6):421–4. 24677148 10.1002/micr.22254

[61] Coriddi M, Dayan J, Bloomfield E, McGrath L, Diwan R, Monge J, et al. Efficacy of Immediate Lymphatic Reconstruction to Decrease Incidence of Breast Cancer-related Lymphedema: Preliminary Results of Randomized Controlled Trial. Ann Surg. 2023;278(4):630–7. 37314177 10.1097/SLA.0000000000005952 PMC10527595

[62] Brorson H. Liposuction in Lymphedema Treatment. J Reconstr Microsurg. 2016;32(1):56–65. 25893630 10.1055/s-0035-1549158

[63] McLaughlin SA, Staley AC, Vicini F, Thiruchelvam P, Hutchison NA, Mendez J, et al. Considerations for Clinicians in the Diagnosis, Prevention, and Treatment of Breast Cancer-Related Lymphedema: Recommendations from a Multidisciplinary Expert ASBrS Panel : Part 1: Definitions, Assessments, Education, and Future Directions. Ann Surg Oncol. 2017;24(10):2818–26. 28766232 10.1245/s10434-017-5982-4

[64] Rafn BS, Christensen J, Larsen A, Bloomquist K. Prospective Surveillance for Breast Cancer-Related Arm Lymphedema: A Systematic Review and Meta-Analysis. J Clin Oncol. 2022;40(9):1009–26. 35077194 10.1200/JCO.21.01681

[65] Rockson SG. Lymphedema after Breast Cancer Treatment. N Engl J Med. 2018;379(20):1937–44. 30428297 10.1056/NEJMcp1803290

[66] version 5.2024. https://www.nccn.org/guidelines/guidelines-detail?category=1&id=1419. Accessed 22 Apr 2025.

[67] Barroso-Sousa R BW, Garrido-Castro AC, Hodi FS, Min L, Krop IE, et al. Incidence of Endocrine Dysfunction Following the Use of Different Immune Checkpoint Inhibitor Regimens. JAMA Oncol. 2018;1;4(2):173. 10.1001/jamaoncol.2017.3064 PMC5838579 28973656

[68] Gao R, Li C, Zhang S, Liao N, Guan H. Immune checkpoint inhibitors-related endocrinopathies exhibit increased severity in breast cancer patients: a real-world study. Endocrine. 2024;85(3):1446–55. 38951449 10.1007/s12020-024-03859-4

[69] Geisler AN, Phillips GS, Barrios DM, Wu J, Leung DYM, Moy AP, et al. Immune checkpoint inhibitor-related dermatologic adverse events. J Am Acad Dermatol. 2020;83(5):1255–68. 32454097 10.1016/j.jaad.2020.03.132 PMC7572894

[70] Sadik CD, Langan EA, Gutzmer R, Fleischer MI, Loquai C, Reinhardt L, et al. Retrospective Analysis of Checkpoint Inhibitor Therapy-Associated Cases of Bullous Pemphigoid From Six German Dermatology Centers. Front Immunol. 2020;11: 588582. 33708189 10.3389/fimmu.2020.588582 PMC7940359

[71] Aggarwal P, Clark D, Shah A, Rismiller K, Neltner SA. Keratoacanthoma and Cutaneous Squamous Cell Carcinoma With PD-1 and PD-L1 Inhibitor Use. JAMA Dermatol. 2024;160(5):573–5. 38598190 10.1001/jamadermatol.2024.0390 PMC11007655

[72] Naidoo J, Wang X, Woo KM, Iyriboz T, Halpenny D, Cunningham J, et al. Pneumonitis in Patients Treated With Anti-Programmed Death-1/Programmed Death Ligand 1 Therapy. J Clin Oncol. 2017;35(7):709–17. 27646942 10.1200/JCO.2016.68.2005 PMC5559901

[73] Baba T, Sakai F, Kato T, Kusumoto M, Kenmotsu H, Sugiura H, et al. Radiologic features of pneumonitis associated with nivolumab in non-small-cell lung cancer and malignant melanoma. Future Oncol. 2019;15(16):1911–20. 31020849 10.2217/fon-2019-0102

[74] Gumusay O, Callan J, Rugo HS. Immunotherapy toxicity: identification and management. Breast Cancer Res Treat. 2022;192(1):1–17. 35015209 10.1007/s10549-021-06480-5 PMC8841316

[75] Dolladille C, Akroun J, Morice PM, Dompmartin A, Ezine E, Sassier M, et al. Cardiovascular immunotoxicities associated with immune checkpoint inhibitors: a safety meta-analysis. Eur Heart J. 2021;42(48):4964–77. 34529770 10.1093/eurheartj/ehab618

[76] Laroche F, Coste J, Medkour T, Cottu PH, Pierga JY, Lotz JP, et al. Classification of and risk factors for estrogen deprivation pain syndromes related to aromatase inhibitor treatments in women with breast cancer: a prospective multicenter cohort study. J Pain. 2014;15(3):293–303. 24365325 10.1016/j.jpain.2013.11.004

[77] Partridge AH, LaFountain A, Mayer E, Taylor BS, Winer E, Asnis-Alibozek A. Adherence to initial adjuvant anastrozole therapy among women with early-stage breast cancer. J Clin Oncol. 2008;26(4):556–62. 18180462 10.1200/JCO.2007.11.5451

[78] Hershman DL, Shao T, Kushi LH, Buono D, Tsai WY, Fehrenbacher L, et al. Early discontinuation and non-adherence to adjuvant hormonal therapy are associated with increased mortality in women with breast cancer. Breast Cancer Res Treat. 2011;126(2):529–37. 20803066 10.1007/s10549-010-1132-4 PMC3462663

[79] Henry NL, Unger JM, Schott AF, Fehrenbacher L, Flynn PJ, Prow DM, et al. Randomized, Multicenter, Placebo-Controlled Clinical Trial of Duloxetine Versus Placebo for Aromatase Inhibitor-Associated Arthralgias in Early-Stage Breast Cancer: SWOG S1202. J Clin Oncol. 2018;36(4):326–32. 29136387 10.1200/JCO.2017.74.6651 PMC5805479

[80] Briot K, Tubiana-Hulin M, Bastit L, Kloos I, Roux C. Effect of a switch of aromatase inhibitors on musculoskeletal symptoms in postmenopausal women with hormone-receptor-positive breast cancer: the ATOLL (articular tolerance of letrozole) study. Breast Cancer Res Treat. 2010;120(1):127–34. 20035381 10.1007/s10549-009-0692-7

[81] Roberts K, Rickett K, Greer R, Woodward N. Management of aromatase inhibitor induced musculoskeletal symptoms in postmenopausal early Breast cancer: A systematic review and meta-analysis. Crit Rev Oncol Hematol. 2017;111:66–80. 28259297 10.1016/j.critrevonc.2017.01.010

[82] Arem H, Sorkin M, Cartmel B, Fiellin M, Capozza S, Harrigan M, et al. Exercise adherence in a randomized trial of exercise on aromatase inhibitor arthralgias in breast cancer survivors: the Hormones and Physical Exercise (HOPE) study. J Cancer Surviv. 2016;10(4):654–62. 26782031 10.1007/s11764-015-0511-6 PMC5418660

[83] Hershman DL, Unger JM, Greenlee H, Capodice JL, Lew DL, Darke AK, et al. Effect of Acupuncture vs Sham Acupuncture or Waitlist Control on Joint Pain Related to Aromatase Inhibitors Among Women With Early-Stage Breast Cancer: A Randomized Clinical Trial. JAMA. 2018;320(2):167–76. 29998338 10.1001/jama.2018.8907 PMC6583520

[84] Rivera DR, Ganz PA, Weyrich MS, Bandos H, Melnikow J. Chemotherapy-Associated Peripheral Neuropathy in Patients With Early-Stage Breast Cancer: A Systematic Review. JNCI: J Nat Cancer Inst. 2018;110(2):djx140. 10.1093/jnci/djx140 PMC5825681 28954296

[85] Kamgar M, Greenwald MK, Assad H, Hastert TA, McLaughlin EM, Reding KW, et al. Prevalence and predictors of peripheral neuropathy after breast cancer treatment. Cancer Med. 2021;10(19):6666–76. 34390205 10.1002/cam4.4202 PMC8495292

[86] Argyriou AA, Bruna J, Marmiroli P, Cavaletti G. Chemotherapy-induced peripheral neurotoxicity (CIPN): An update. Crit Rev Oncol Hematol. 2012;82(1):51–77. 21908200 10.1016/j.critrevonc.2011.04.012

[87] Molinares D, Kurtevski S, Zhu Y. Chemotherapy-Induced Peripheral Neuropathy: Diagnosis, Agents, General Clinical Presentation, and Treatments. Curr Oncol Rep. 2023;25(11):1227–35. 37702983 10.1007/s11912-023-01449-7

[88] Zukas AM, Schiff D. Neurological complications of new chemotherapy agents. Neuro Oncol. 2018;20(1):24–36. 28992326 10.1093/neuonc/nox115 PMC5761528

[89] Markides DM, Hita AG, Merlin J, Reyes-Gibby C, Yeung SJ. Antibody-drug conjugates: the toxicities and adverse effects that emergency physicians must know. Ann Emerg Med. 2025;85(3):214–29. 10.1016/j.annemergmed.2024.10.015. 10.1016/j.annemergmed.2024.10.015 39641680

[90] Flatters SJL, Dougherty PM, Colvin LA. Clinical and preclinical perspectives on Chemotherapy-Induced Peripheral Neuropathy (CIPN): a narrative review. Br J Anaesth. 2017;119(4):737–49. 29121279 10.1093/bja/aex229

[91] Cavaletti G, Alberti P, Frigeni B, Piatti M, Susani E. Chemotherapy-Induced Neuropathy. Curr Treat Options Neurol. 2011;13(2):180–90. 21191824 10.1007/s11940-010-0108-3

[92] Bhatnagar B, Gilmore S, Goloubeva O, Pelser C, Medeiros M, Chumsri S, et al. Chemotherapy dose reduction due to chemotherapy induced peripheral neuropathy in breast cancer patients receiving chemotherapy in the neoadjuvant or adjuvant settings: a single-center experience. Springerplus. 2014;3(1):366. 25089251 10.1186/2193-1801-3-366 PMC4117856

[93] Engvall K, Gréen H, Fredrikson M, Lagerlund M, Lewin F, Åvall-Lundqvist E. Impact of persistent peripheral neuropathy on health-related quality of life among early-stage breast cancer survivors: a population-based cross-sectional study. Breast Cancer Res Treat. 2022;195(3):379–91. 35941422 10.1007/s10549-022-06670-9 PMC9464756

[94] Mustafa Ali M, Moeller M, Rybicki L, Moore HCF. Long-term peripheral neuropathy symptoms in breast cancer survivors. Breast Cancer Res Treat. 2017;166(2):519–26. 28791499 10.1007/s10549-017-4437-8

[95] Greenlee H, Hershman DL, Shi Z, Kwan ML, Ergas IJ, Roh JM, et al. BMI, Lifestyle Factors and Taxane-Induced Neuropathy in Breast Cancer Patients: The Pathways Study. JNCI: J Nat Cancer Inst. 2017;109(2):djw206. 10.1093/jnci/djw206 PMC6093415 27794123

[96] Song SJ, Min J, Suh SY, Jung SH, Hahn HJ, Im S-A, et al. Incidence of taxane-induced peripheral neuropathy receiving treatment and prescription patterns in patients with breast cancer. Support Care Cancer. 2017;25(7):2241–8. 28204996 10.1007/s00520-017-3631-x

[97] Schneider BP, Zhao F, Wang M, Stearns V, Martino S, Jones V, et al. Neuropathy Is Not Associated With Clinical Outcomes in Patients Receiving Adjuvant Taxane-Containing Therapy for Operable Breast Cancer. J Clin Oncol. 2012;30(25):3051–7. 22851566 10.1200/JCO.2011.39.8446 PMC3732004

[98] Molassiotis A, Cheng HL, Lopez V, Au JSK, Chan A, Bandla A, et al. Are we mis-estimating chemotherapy-induced peripheral neuropathy? Analysis of assessment methodologies from a prospective, multinational, longitudinal cohort study of patients receiving neurotoxic chemotherapy. BMC Cancer. 2019;19(1):132. 30736741 10.1186/s12885-019-5302-4 PMC6368751

[99] Schneider BP, Lai D, Shen F, Jiang G, Radovich M, Li L, Gardner L, Miller KD, O'Neill A, Sparano JA, Xue G, Foroud T, Sledge GW Jr. Charcot-Marie-Tooth gene, SBF2, associated with taxane-induced peripheral neuropathy in African Americans. Oncotarget. 2016;7(50):82244–53. 10.18632/oncotarget.12545. 10.18632/oncotarget.12545 PMC5347688 27732968

[100] Schneider BP, Li L, Radovich M, Shen F, Miller KD, Flockhart DA, et al. Genome-Wide Association Studies for Taxane-Induced Peripheral Neuropathy in ECOG-5103 and ECOG-1199. Clin Cancer Res. 2015;21(22):5082–91. 26138065 10.1158/1078-0432.CCR-15-0586 PMC4717479

[101] Zajączkowska R, Kocot-Kępska M, Leppert W, Wrzosek A, Mika J, Wordliczek J. Mechanisms of chemotherapy-induced peripheral neuropathy. Int J Mol Sci. 2019;20(6):1451. 10.3390/ijms20061451. 10.3390/ijms20061451 PMC6471666 30909387

[102] Nyrop KA, Deal AM, Reeder-Hayes KE, Shachar SS, Reeve BB, Basch E, et al. Patient-reported and clinician-reported chemotherapy-induced peripheral neuropathy in patients with early breast cancer: Current clinical practice. Cancer. 2019;125(17):2945–54. 31090930 10.1002/cncr.32175

[103] Loprinzi CL, Reeves BN, Dakhil SR, Sloan JA, Wolf SL, Burger KN, et al. Natural History of Paclitaxel-Associated Acute Pain Syndrome: Prospective Cohort Study NCCTG N08C1. J Clin Oncol. 2011;29(11):1472–8. 21383290 10.1200/JCO.2010.33.0308 PMC3082985

[104] Kober KM, Lee M-C, Olshen A, Conley YP, Sirota M, Keiser M, et al. Differential methylation and expression of genes in the hypoxia-inducible factor 1 signaling pathway are associated with paclitaxel-induced peripheral neuropathy in breast cancer survivors and with preclinical models of chemotherapy-induced neuropathic pain. Mol Pain. 2020;16:1744806920936502. 32586194 10.1177/1744806920936502 PMC7322824

[105] Mortensen C, Thomsen MT, Chua KC, Hammer HS, Nielsen F, Pötz O, et al. Modeling mechanisms of chemotherapy-induced peripheral neuropathy and chemotherapy transport using induced pluripotent stem cell-derived sensory neurons. Neuropharmacology. 2024;258: 110062. 38972371 10.1016/j.neuropharm.2024.110062

[106] Areti A, Yerra VG, Naidu VGM, Kumar A. Oxidative stress and nerve damage: Role in chemotherapy induced peripheral neuropathy. Redox Biol. 2014;2:289–95. 24494204 10.1016/j.redox.2014.01.006 PMC3909836

[107] Doyle TM, Janes K, Xiao WH, Kolar GR, Luecke HF, Gratton MA, Tosh DK, Jacobson KA, Bennett GJ, Salvemini D. Mitochondrial A3 adenosine receptor as a mechanism for the protective effects of A3AR agonists on chemotherapy-induced neuropathic pain. J Neurosci. 2025;45(3):e1268242024. 10.1523/JNEUROSCI.1268-24.2024. 10.1523/JNEUROSCI.1268-24.2024 PMC11735668 39653498

[108] Hassanzadeh E, Sedighi Pashaki A, Akbari Hamed E, Mehrpooya M, Mohammadian K, Bayani R, Sheikhi K, Ranjbar H, Abbasi M. Evaluating N-acetylcysteine as a protective agent against chemotherapy-induced neuropathy in breast cancer: a triple-blind, randomized clinical trial. Am J Clin Oncol. 2025;48(3):122–6. 10.1097/COC.0000000000001153. 10.1097/COC.0000000000001153 39494844

[109] Chen J, Li H. Characterization of novel SARM1 inhibitors for the treatment of chemotherapy-induced peripheral neuropathy. Biomedicines. 2024;12:2123. 10.3390/biomedicines12092123. 10.3390/biomedicines12092123 PMC11428815 39335636

[110] Sun X, Huang W, Yin D, Zhao X, Cheng X, Zhang J, et al. Nicotinamide riboside activates SIRT3 to prevent paclitaxel-induced peripheral neuropathy. Toxicol Appl Pharmacol. 2024;491: 117066. 39128506 10.1016/j.taap.2024.117066

[111] Janes K, Little JW, Li C, Bryant L, Chen C, Chen Z, et al. The development and maintenance of paclitaxel-induced neuropathic pain require activation of the sphingosine 1-phosphate receptor subtype 1. J Biol Chem. 2014;289(30):21082–97. 24876379 10.1074/jbc.M114.569574 PMC4110312

[112] Anukriti Sharma KBJ, Alper Sen, Bihua Bie, Emily E. Rhoades, Courtney Hershberger, Mei Wei, N. Lynn Henry, Carla Bou Dargham, G. Thomas Budd, Joseph Foss, Daniel M. Rotroff Longitudinal multi-omics study reveals potential targets against chemotherapy induced peripheral neuropathy during taxane treatment SABCS abstract Number: P1–04–02. 2024. https://sabcs.org/Portals/0/Documents/FINAL%20SABCS%2024%20%20Poster%20Session%201%20.pdf?ver=COtfEZSxfP5lWkHrNkFAqg%3D%3D.

[113] Bandos H, Melnikow J, Rivera DR, Swain SM, Sturtz K, Fehrenbacher L, et al. Long-term Peripheral Neuropathy in Breast Cancer Patients Treated With Adjuvant Chemotherapy: NRG Oncology/NSABP B-30. JNCI: J Nat Cancer Inst. 2018;110(2):djx162. 10.1093/jnci/djx162 PMC5825682 28954297

[114] Swain SM, Jeong J-H, Geyer CE, Costantino JP, Pajon ER, Fehrenbacher L, et al. Longer Therapy, Iatrogenic Amenorrhea, and Survival in Early Breast Cancer. N Engl J Med. 2010;362(22):2053–65. 20519679 10.1056/NEJMoa0909638 PMC2935316

[115] Loprinzi CL, Lacchetti C, Bleeker J, Cavaletti G, Chauhan C, Hertz DL, et al. Prevention and Management of Chemotherapy-Induced Peripheral Neuropathy in Survivors of Adult Cancers: ASCO Guideline Update. J Clin Oncol. 2020;38(28):3325–48. 32663120 10.1200/JCO.20.01399

[116] Wan Z, Huang J, Wang X, Li P. Compression Therapy for Prevention of Chemotherapy-Induced Peripheral Neuropathy: A Systematic Review and Meta-Analysis of Randomized Controlled Trials. J Pain Res. 2024;17:4509–19. 39735655 10.2147/JPR.S488470 PMC11681779

[117] Okazaki M, Bando H, Terasaki A, Ueda A, Iguchi-Manaka A, Mathis BJ, et al. Safety and Efficacy of Compression Therapy to Prevent Chemotherapy-Induced Peripheral Neuropathy in Lower Extremities of Breast Cancer Patients: A Pilot Study. Cureus. 2024;16(5): e60998. 38910688 10.7759/cureus.60998 PMC11193973

[118] Accordino MK, Lee S, Leu CS, Levin B, Trivedi MS, Crew KD, et al. Randomized adaptive selection trial of cryotherapy, compression therapy, and placebo to prevent taxane-induced peripheral neuropathy in patients with breast cancer. Breast Cancer Res Treat. 2024;204(1):49–59. 38060077 10.1007/s10549-023-07172-y PMC10840989

[119] Dongxue G, Ran L, Fangfei Z, Zirui Z, Lizhi Z. Therapeutic effects of compression therapy on taxane-induced peripheral neuropathy incidence, negative emotions, and sleep disorders in patients with breast cancer. Support Care Cancer. 2024;32(4):260. 38561474 10.1007/s00520-024-08461-y

[120] Johnson K, Stoffel B, Schwitter M, Hayoz S, Rojas Mora A, Fischer Maranta A, et al. Prevention of taxane chemotherapy-induced nail changes and peripheral neuropathy by application of extremity cooling: a prospective single-centre study with intrapatient comparison. Support Care Cancer. 2024;32(8):554. 39066890 10.1007/s00520-024-08737-3 PMC11283420

[121] Tai HY, Lin LY, Huang TW, Gautama MSN. Efficacy of cryotherapy in the prevention of chemotherapy-induced peripheral neuropathy: A systematic review and meta-analysis. Support Care Cancer. 2024;32(7):482. 38955817 10.1007/s00520-024-08680-3

[122] Lendorf ME. Effects of cryotherapy on objective and subjective symptoms of taxane induced neuropathy in patients with early breast cancer: a national, multicenter, prospective, randomized, controlled trial. SABCS Abtract Number: 1-PS5–08. 2024. https://sabcs.org/Portals/0/Documents/FINAL%20SABCS%2024%20%20Poster%20Session%201%20.pdf?ver=COtfEZSxfP5lWkHrNkFAqg%3D%3D.

[123] Brunner C, Emmelheinz M, Egle D, Ritter M, Leitner K, Wieser V, et al. Cropsi study: Efficacy and safety of cryotherapy and cryocompression in the prevention of chemotherapy-induced peripheral neuropathy in patients with breast and gynecological cancer-A prospective, randomized trial. Breast. 2024;76: 103763. 38941655 10.1016/j.breast.2024.103763 PMC11260371

[124] Yao Y, Zhang X, Ge R, Im HS, Yao C. Electroacupuncture for the prevention of chemotherapy-induced peripheral neuropathy: a randomized controlled trial. Zhongguo Zhen Jiu. 2024;44(12):1388–94. 39658376 10.13703/j.0255-2930.20240222-k0001

[125] Huang MC, Chang SC, Liao WL, Ke TW, Lee AL, Wang HM, et al. Acupuncture May Help to Prevent Chemotherapy-Induced Peripheral Neuropathy: A Randomized, Sham-Controlled. Single-Blind Study Oncologist. 2023;28(6):e436–47. 36971468 10.1093/oncolo/oyad065 PMC10243779

[126] Aghili M, Taherioun M, Jafari F, Azadvari M, Lashkari M, Kolahdouzan K, et al. Duloxetine to prevent neuropathy in breast cancer patients under paclitaxel chemotherapy (a double-blind randomized trial). Support Care Cancer. 2024;32(8):493. 38976095 10.1007/s00520-024-08669-y

[127] Joy L, Jolien R, Marithé C, Stijn E, Laura S, Hilde L, et al. The use of photobiomodulation therapy for the prevention of chemotherapy-induced peripheral neuropathy: a randomized, placebo-controlled pilot trial (NEUROLASER trial). Support Care Cancer. 2022;30(6):5509–17. 35312857 10.1007/s00520-022-06975-x PMC8935622

[128] Kang YJ, Yoon CI, Yang YJ, Baek JM, Kim YS, Jeon YW, et al. A randomized controlled trial using surgical gloves to prevent chemotherapy-induced peripheral neuropathy by paclitaxel in breast cancer patients (AIUR trial). BMC Cancer. 2023;23(1):570. 37340369 10.1186/s12885-023-11079-8 PMC10283240

[129] Smith EM, Pang H, Cirrincione C, Fleishman S, Paskett ED, Ahles T, et al. Effect of duloxetine on pain, function, and quality of life among patients with chemotherapy-induced painful peripheral neuropathy: a randomized clinical trial. JAMA. 2013;309(13):1359–67. 23549581 10.1001/jama.2013.2813 PMC3912515

[130] Meng J, Zhang Q, Yang C, Xiao L, Xue Z, Zhu J. Duloxetine, a Balanced Serotonin-Norepinephrine Reuptake Inhibitor, Improves Painful Chemotherapy-Induced Peripheral Neuropathy by Inhibiting Activation of p38 MAPK and NF-κB. Front Pharmacol. 2019;10:365. 31024320 10.3389/fphar.2019.00365 PMC6465602

[131] Velasco R, Besora S, Argyriou AA, Santos C, Sala R, Izquierdo C, et al. Duloxetine against symptomatic chemotherapy-induced peripheral neurotoxicity in cancer survivors: a real world, open-label experience. Anticancer Drugs. 2021;32(1):88–94. 33332891 10.1097/CAD.0000000000001005

[132] Kleckner IR, Kamen C, Gewandter JS, Mohile NA, Heckler CE, Culakova E, et al. Effects of exercise during chemotherapy on chemotherapy-induced peripheral neuropathy: a multicenter, randomized controlled trial. Support Care Cancer. 2018;26(4):1019–28. 29243164 10.1007/s00520-017-4013-0 PMC5823751

[133] Nuñez de Arenas-Arroyo S, Cavero-Redondo I, Torres-Costoso A, Reina-Gutiérrez S, Lorenzo-García P, Martínez-Vizcaíno V. Effects of exercise interventions to reduce chemotherapy-induced peripheral neuropathy severity: A meta-analysis. Scand J Med Sci Sports. 2023;33(7):1040–53. 36972017 10.1111/sms.14360

[134] Huang Y, Tan T, Liu L, Yan Z, Deng Y, Li G, Li M, Xiong J. Exercise for reducing chemotherapy-induced peripheral neuropathy: a systematic review and meta-analysis of randomized controlled trials. Front Neurol. 2024;14:1252259. 10.3389/fneur.2023.1252259. Erratum in: Front Neurol. 2024;15:1378461. 10.3389/fneur.2024.1378461. Erratum in: Front Neurol. 2024;15:1460992. 10.3389/fneur.2024.1460992. 10.3389/fneur.2023.1252259 PMC10813204 38283674

[135] Kleckner IR, Manuweera T, Lin PJ, Chung KH, Kleckner AS, Gewandter JS, et al. Pilot trial testing the effects of exercise on chemotherapy-induced peripheral neurotoxicity (CIPN) and the interoceptive brain system. Support Care Cancer. 2024;32(10):677. 39304604 10.1007/s00520-024-08855-y PMC12203810

[136] Li RL, Liu JE, Bai LX, Yang AL, Liu Y, Zhao FY, et al. Striding beyond numbness: a non-randomized controlled study of an exercise program for breast cancer patients with chemotherapy-induced peripheral neuropathy. Support Care Cancer. 2024;32(12):837. 39611862 10.1007/s00520-024-09003-2

[137] Lu C, Feng X, Shen Q, Li G, Wu T, Li X, et al. Electroacupuncture with different frequencies for paclitaxel-induced peripheral neuropathy: a randomized controlled trial. Zhongguo Zhen Jiu. 2024;44(10):1139–45. 39401811 10.13703/j.0255-2930.20240123-0001

[138] Lu W, Giobbie-Hurder A, Freedman RA, Shin IH, Lin NU, Partridge AH, et al. Acupuncture for Chemotherapy-Induced Peripheral Neuropathy in Breast Cancer Survivors: A Randomized Controlled Pilot Trial. Oncologist. 2020;25(4):310–8. 32297442 10.1634/theoncologist.2019-0489 PMC7160396

[139] Knoerl R, Giobbie-Hurder A, Berfield J, Berry D, Meyerhardt JA, Wright AA, et al. Yoga for chronic chemotherapy-induced peripheral neuropathy pain: a pilot, randomized controlled trial. J Cancer Surviv. 2022;16(4):882–91. 34524631 10.1007/s11764-021-01081-z PMC8442518

[140] A Randomized Phase III Clinical Trial of Yoga for Chemotherapy-induced Peripheral Neuropathy Treatment (YCT) [Internet]. 2021. Available from: https://clinicaltrials.gov/study/NCT05121558. Accessed 22 Apr 2025.

[141] A Randomized Phase III Clinical Trial of Acupuncture for Chemotherapy-induced Peripheral Neuropathy Treatment (ACT) [Internet]. 2021. Available from: https://clinicaltrials.gov/study/NCT04917796. Accessed 22 Apr 2025.

[142] Ran L, Dongxue G, Zirui Z, Jiwei H, Aijun D, Yuchen H, et al. Effects of intermittent hand-foot hypothermia therapy on chemotherapy-induced peripheral neurotoxicity. Support Care Cancer. 2024;33(1):43. 39702596 10.1007/s00520-024-09065-2

[143] Misawa S, Denda T, Kodama S, Suzuki T, Naito Y, Kogawa T, et al. Efficacy and safety of mirogabalin for chemotherapy-induced peripheral neuropathy: a prospective single-arm trial (MiroCIP study). BMC Cancer. 2023;23(1):1098. 37951905 10.1186/s12885-023-11560-4 PMC10640752

[144] Aghili M, Zare M, Mousavi N, Ghalehtaki R, Sotoudeh S, Kalaghchi B, et al. Efficacy of gabapentin for the prevention of paclitaxel induced peripheral neuropathy: A randomized placebo controlled clinical trial. Breast J. 2019;25(2):226–31. 30773731 10.1111/tbj.13196

[145] Rao RD, Michalak JC, Sloan JA, Loprinzi CL, Soori GS, Nikcevich DA, et al. Efficacy of gabapentin in the management of chemotherapy-induced peripheral neuropathy: a phase 3 randomized, double-blind, placebo-controlled, crossover trial (N00C3). Cancer. 2007;110(9):2110–8. 17853395 10.1002/cncr.23008

[146] Prinsloo S, Kaptchuk TJ, De Ridder D, Lyle R, Bruera E, Novy D, et al. Brain-computer interface relieves chronic chemotherapy-induced peripheral neuropathy: A randomized, double-blind, placebo-controlled trial. Cancer. 2024;130(2):300–11. 37733286 10.1002/cncr.35027

[147] Pharmacokinetics and Safety of Epidiferphane and Taxanes in Breast Cancer Patients [Internet]. 2021. Available from: https://clinicaltrials.gov/study/NCT05074290. Accessed 22 Apr 2025.

[148] Sonia Servitja Maria Castro-Henriques IÁ-B, Miguel Servet, Carlota Díez-Franco, Alba Medina-Castillo, Maria Asunción Algarra-García, Marina Baixa, Villajoyosa, Alicante, Elena López-Miranda, Ramon y Cajal, Margaret Lario-Martínez, Ramon y Cajal, Maria Isabel Luengo-Alcázar, Miguel Borregón, Ana Davó, Elche, Alicante, Anna Gassull-Delgado, Sara Roque-García, Ana Gonzaga-López, Elda, Alicante, Jesus Manuel Poveda-Ferriols, Severine Pascal, Clotilde Ferrándiz-Huertas, Miguel Hernández, Ana María Mitroi-Marinescu, Marta García-Escolano, Miguel Hernández, Fernández-Carvajal, Miguel Hernández, Antonio Ferrer Montiel. Topical modulation of nociceptor epidermal terminals delays and ameliorates CIPN improving the quality of life of breast cancer patients. SABCS abstract Number: P1–02–07. 2024. https://sabcs.org/Portals/0/Documents/FINAL%20SABCS%2024%20%20Poster%20Session%201%20.pdf?ver=COtfEZSxfP5lWkHrNkFAqg%3D%3D.

[149] Li T, Timmins HC, Mahfouz FM, Trinh T, Mizrahi D, Horvath LG, et al. Validity of Patient-Reported Outcome Measures in Evaluating Nerve Damage Following Chemotherapy. JAMA Network Open. 2024;7(8):e2424139-e. 10.1001/jamanetworkopen.2024.24139 PMC11316238 39120903

[150] Burton LB, Eskian M, Guidon AC, Reynolds KL. A review of neurotoxicities associated with immunotherapy and a framework for evaluation. Neuro-Oncology Advances. 2021;3(Supplement_5):v108-v20. 10.1093/noajnl/vdab107 PMC8633791 34859238

[151] Haugh AM, Probasco JC, Johnson DB. Neurologic complications of immune checkpoint inhibitors. Expert Opin Drug Saf. 2020;19(4):479–88. 32126176 10.1080/14740338.2020.1738382 PMC7192781

[152] DeMichele A, Clark AS, Tan KS, Heitjan DF, Gramlich K, Gallagher M, et al. CDK 4/6 Inhibitor Palbociclib (PD0332991) in Rb+ Advanced Breast Cancer: Phase II Activity, Safety, and Predictive Biomarker Assessment. Clin Cancer Res. 2015;21(5):995–1001. 25501126 10.1158/1078-0432.CCR-14-2258

[153] Harris SR. Brachial plexopathy after breast cancer: A persistent late effect of radiotherapy. PM&R. 2024;16(1):85–91. 37272709 10.1002/pmrj.13007

[154] Pradat P-F, Delanian S. Chapter 43 - Late radiation injury to peripheral nerves. In: Said G, Krarup C, editors. Handbook of Clinical Neurology. 115: Elsevier; 2013. p. 743–58. 10.1016/B978-0-444-52902-2.00043-6 23931813

[155] Avila F, Torres-Guzman R, Maita K, Garcia JP, De Sario GD, Borna S, et al. A Review on the Management of Peripheral Neuropathic Pain Following Breast Cancer. Breast Cancer (Dove Med Press). 2023;15:761–72. 37927491 10.2147/BCTT.S386803 PMC10624189

[156] Waltho D, Rockwell G. Post–breast surgery pain syndrome: establishing a consensus for the definition of post-mastectomy pain syndrome to provide a standardized clinical and research approach — a review of the literature and discussion. Can J Surg. 2016;59(5):342–50. 27668333 10.1503/cjs.000716 PMC5042722

[157] Stubblefield MD, Custodio CM. Upper-Extremity Pain Disorders in Breast Cancer. Arch Phys Med Rehabil. 2006;87(3):96–9. 16500198 10.1016/j.apmr.2005.12.017

[158] Medford AJMB, Spring LM, Hurvitz SA, Turner NC, Bardia A. Molecular residual disease in breast cancer: detection and therapeutic interception. Clin Cancer Res. 2023;29(22):4540–8. 37477704 10.1158/1078-0432.CCR-23-0757

[159] Garcia-Murillas I SG, Weigelt B, Ng C, Hrebien S, Cutts RJ, Cheang M, Osin P, Nerurkar A, Kozarewa I, Garrido JA. Mutation tracking in circulating tumor DNA predicts relapse in early breast cancer. Science translational medicine. 2015 7(302):302ra133-. 10.1126/scitranslmed.aab0021 26311728

[160] Gale D HK, Ruiz-Valdepenas A, Hackinger S, Perry M, Marsico G, Rundell V, Wulff J, Sharma G, Knock H, Castedo. Residual ctDNA after treatment predicts early relapse in patients with early-stage non-small cell lung cancer. J Annals of oncology. 2022;33(5):500–10. 10.1016/j.annonc.2022.02.007 PMC9067454 35306155

[161] Reinert THT, Christensen E, Sharma S, Salari R, Sethi H, Knudsen M, Nordentoft I, Wu HT, Tin AS, Rasmussen MH. Analysis of plasma cell-free DNA by ultradeep sequencing in patients with stages I to III colorectal cancer. JAMA Oncol. 2019;5(8):1124–31. 31070691 10.1001/jamaoncol.2019.0528 PMC6512280

[162] Stecklein SRKB, Yoder R, Schwensen K, Staley JM, Khan QJ, O’Dea AP, Nye LE, Elia M, Heldstab J, Home T. ctDNA and residual cancer burden are prognostic in triple-negative breast cancer patients with residual disease. NPJ Breast Cancer. 2023;9(1):10. 36878909 10.1038/s41523-023-00512-7 PMC9988835

[163] Butler TMBC, Johnson-Camacho K, Tabatabaei S, Melendez D, Kelley T, Gray J, Corless CL, Spellman PT. Circulating tumor DNA dynamics using patient-customized assays are associated with outcome in neoadjuvantly treated breast cancer. Molecular Case Studies. 2019;5(2): a003772. 30833418 10.1101/mcs.a003772 PMC6549569

[164] Garcia-Murillas I, et al. Whole genome sequencing-powered ctDNA sequencing for breast cancer detection. Ann Oncol. 36(6):673–81. 10.1016/j.annonc.2025.01.021 39914664

[165] Mergel F, Huesmann ST, Friedl TWP, Pfister K. A multicenter, randomized, controlled phase 3 superiority trial, evaluating liquid biopsy-guided intensified follow-up surveillance in women with breast cancer. San Antonio Breast Cancer Symposium. 2023.

[166] Welch HG, Dossett LA. Routine surveillance for cancer metastases - does it help or harm patients? N Engl J Med. 2025;392(17):1667–70. 10.1056/NEJMp2414159. 10.1056/NEJMp2414159 40293173

[167] Shapiro CL, Van Poznak C, Lacchetti C, Kirshner J, Eastell R, Gagel R, Smith S, Edwards BJ, Frank E, Lyman GH, Smith MR, Mhaskar R, Henderson T, Neuner J. Management of osteoporosis in survivors of adult cancers with nonmetastatic disease: ASCO clinical practice guideline. J Clin Oncol. 2019;37(31):2916–46. 10.1200/JCO.19.01696. 10.1200/JCO.19.01696 31532726

[168] Coleman RE, Bolten WW, Lansdown M, Dale S, Jackisch C, Merkel D, et al. Aromatase inhibitor-induced arthralgia: clinical experience and treatment recommendations. Cancer Treat Rev. 2008;34(3):275–82. 18082328 10.1016/j.ctrv.2007.10.004

[169] Amir ESB, Niraula S, Carlsson L, Ocaña A. Toxicity of adjuvant endocrine therapy in postmenopausal breast cancer patients: a systematic review and meta-analysis. J Natl Cancer Inst. 2011;103(17):1299–309. 21743022 10.1093/jnci/djr242

[170] Goldvaser H BT, Šeruga B, Cescon DW, Ocaña A, Ribnikar D, Amir E. Toxicity of extended adjuvant therapy with aromatase inhibitors in early breast cancer: a systematic review and meta-analysis. JNCI: J Nat Cancer Inst 2018;110 (1):31–9 10.1093/jnci/djx141 28922781

[171] Yu Z, Sun D, Sun J. Social Support and Fear of Cancer Recurrence Among Chinese Breast Cancer Survivors: The Mediation Role of Illness Uncertainty. Front Psychol. 2022; 13 - 2022. 10.3389/fpsyg.2022.864129 PMC8966644 35369168

[172] Luigjes-Huizer YL, van der Lee ML, Richel C, Masselink RA, de Wit NJ, Helsper CW. Patient-reported needs for coping with worry or fear about cancer recurrence and the extent to which they are being met: a survey study. J Cancer Surviv. 2024;18(3):791–9. 10.1007/s11764-022-01326-5. 10.1007/s11764-022-01326-5 PMC9803402 36585574

[173] Fan R, Wang L, Bu X, Wang W, Zhu J. Unmet supportive care needs of breast cancer survivors: a systematic scoping review. BMC Cancer. 2023;23(1):587. 37365504 10.1186/s12885-023-11087-8 PMC10294377

[174] Martínez Arroyo O, Andreu Vaíllo Y, Martínez López P, Galdón Garrido MJ. Emotional distress and unmet supportive care needs in survivors of breast cancer beyond the end of primary treatment. Support Care Cancer. 2019;27(3):1049–57. 30094729 10.1007/s00520-018-4394-8

[175] Runowicz CD, Leach CR, Henry NL, Henry KS, Mackey HT, Cowens-Alvarado RL, et al. American Cancer Society/American Society of Clinical Oncology Breast Cancer Survivorship Care Guideline. J Clin Oncol. 2016;34(6):611–35. 26644543 10.1200/JCO.2015.64.3809

[176] Zarei M, Sharif-Nia H, Lehto RH, Goudarzian AH, Ashghali-Farahani M. Relationship Between Fear of Cancer Recurrence and Quality of Life in Patients Undergoing Active Cancer Treatment: The Mediating Role of Social Support. Asian Pac J Cancer Prev. 2024;25(5):1787–93. 38809651 10.31557/APJCP.2024.25.5.1787 PMC11318823

[177] Jansen BA, Bargon CA, Dinger TL, van den Goor M, Postma EL, Young-Afat DA, et al. Breast cancer patients’ needs and perspectives on a one-on-one peer support program: quantitative and qualitative analyses. Support Care Cancer. 2023;31(12):656. 37882849 10.1007/s00520-023-08009-6 PMC10602952

[178] Elsous A, Radwan M, Najjar S, Masad A, Abu RM. Unmet needs and health-related quality of life of breast cancer survivors: survey from Gaza Strip. Palestine Acta Oncol. 2023;62(2):194–209. 36802358 10.1080/0284186X.2023.2180326

[179] Dai Q, Liu X, Xu X, Fu Y, She Z, Huang Y, et al. Development of a supportive care framework for breast cancer survivor’s unmet needs: A modified Delphi study. J Clin Nurs. 2024;33(4):1376–86. 38356222 10.1111/jocn.16963

[180] Capelan M, Battisti NML, McLoughlin A, Maidens V, Snuggs N, Slyk P, et al. The prevalence of unmet needs in 625 women living beyond a diagnosis of early breast cancer. Br J Cancer. 2017;117(8):1113–20. 28859057 10.1038/bjc.2017.283 PMC5674103

[181] Ligibel JA, Bohlke K, May AM, Clinton SK, Demark-Wahnefried W, Gilchrist SC, et al. Exercise, Diet, and Weight Management During Cancer Treatment: ASCO Guideline. J Clin Oncol. 2022;40(22):2491–507. 35576506 10.1200/JCO.22.00687

[182] Shen S, Liu B, Fanti C, Bromberg M, Chen Y, Chang C, et al. Glucagon-like peptide-1 (GLP-1) agonist use and weight change among patients with breast cancer. J Clin Oncol. 2024;42(16_suppl):10607-.

[183] Fischbach NA, Zhou B, Deng Y, Parsons K, Shelton A, Lustberg MB. Impact of semaglutide and tirzepatide administration on weight in women with stage I-III breast cancer. J Clin Oncol. 2024;42(16_suppl):e24140-e.

[184] Grunvald E, Shah R, Hernaez R, Chandar AK, Pickett-Blakely O, Teigen LM, et al. AGA Clinical Practice Guideline on Pharmacological Interventions for Adults With Obesity. Gastroenterology. 2022;163(5):1198–225. 36273831 10.1053/j.gastro.2022.08.045

[185] Ligumsky H, Amir S, Arbel Rubinstein T, Guion K, Scherf T, Karasik A, et al. Glucagon-like peptide-1 analogs activate AMP kinase leading to reversal of the Warburg metabolic switch in breast cancer cells. Med Oncol. 2024;41(6):138. 38705935 10.1007/s12032-024-02390-w

[186] Iwaya C, Nomiyama T, Komatsu S, Kawanami T, Tsutsumi Y, Hamaguchi Y, et al. Exendin-4, a Glucagonlike Peptide-1 Receptor Agonist, Attenuates Breast Cancer Growth by Inhibiting NF-κB Activation. Endocrinology. 2017;158(12):4218–32. 29045658 10.1210/en.2017-00461

[187] Levy S, Attia A, Elshazli RM, Abdelmaksoud A, Tatum D, Aiash H, et al. Differential Effects of GLP-1 Receptor Agonists on Cancer Risk in Obesity: A Nationwide Analysis of 1.1 Million Patients. Cancers (Basel). 2024;17(1). 10.3390/cancers17010078 PMC11720624 39796706

[188] Sarfraz H, Bari S, Eysha M, Patel K, Dutta T, Gardell S, et al. Safety and efficacy of time restricted eating (Tre) in improving response to immunotherapy in patients with head and neck cancer (Hnscc). Am Soc Clin Oncol. 2023;31:6051. 10.1200/JCO.2023.41.16_suppl.605.

[189] Ivory J, Wheeler SB, Drier S, Gunn H, Zahrieh D, Paskett E, et al. Randomized phase III trial evaluating motivational interviewing and text interventions to optimize adherence to breast cancer endocrine therapy (Alliance A191901): the GETSET protocol. Trials. 2023;24(1):664. 37828596 10.1186/s13063-023-07672-8 PMC10568920

[190] Sanft T, Harrigan M, McGowan C, Cartmel B, Zupa M, Li FY, et al. Randomized Trial of Exercise and Nutrition on Chemotherapy Completion and Pathologic Complete Response in Women With Breast Cancer: The Lifestyle, Exercise, and Nutrition Early After Diagnosis Study. J Clin Oncol. 2023;41(34):5285–95. 37656930 10.1200/JCO.23.00871 PMC10691793

[191] Geyer CE, Blum JL, Yothers G, Asmar L, Flynn PJ, Robert NJ, et al. Long-Term Follow-Up of the Anthracyclines in Early Breast Cancer Trials (USOR 06–090, NSABP B-46-I/USOR 07132, and NSABP B-49 [NRG Oncology]). J Clin Oncol. 2024;42(12):1344–9. 38335467 10.1200/JCO.23.01428 PMC11095853

[192] Dempke WCM, Zielinski R, Winkler C, Silberman S, Reuther S, Priebe W. Anthracycline-induced cardiotoxicity & #x2014; are we about to clear this hurdle? Eur J Cancer. 2023;185:94–104. 36966697 10.1016/j.ejca.2023.02.019

[193] Larsen CM, Garcia Arango M, Dasari H, Arciniegas Calle M, Adjei E, Rico Mesa J, et al. Association of Anthracycline With Heart Failure in Patients Treated for Breast Cancer or Lymphoma, 1985–2010. JAMA Network Open. 2023;6(2):e2254669-e. 10.1001/jamanetworkopen.2022.54669 PMC9898820 36735254

[194] Cardinale D, Iacopo F, Cipolla CM. Cardiotoxicity of Anthracyclines Front Cardiovasc Med. 2020;7:26. 32258060 10.3389/fcvm.2020.00026 PMC7093379

[195] Lyon AR, López-Fernández T, Couch LS, Asteggiano R, Aznar MC, Bergler-Klein J, et al. 2022 ESC Guidelines on cardio-oncology developed in collaboration with the European Hematology Association (EHA), the European Society for Therapeutic Radiology and Oncology (ESTRO) and the International Cardio-Oncology Society (IC-OS): Developed by the task force on cardio-oncology of the European Society of Cardiology (ESC). Eur Heart J. 2022;43(41):4229–361. 36017568 10.1093/eurheartj/ehac244

[196] Koppelmans V, Breteler MM, Boogerd W, Seynaeve C, Gundy C, Schagen SB. Neuropsychological performance in survivors of breast cancer more than 20 years after adjuvant chemotherapy. J Clin Oncol. 2012;30(10):1080–6. 22370315 10.1200/JCO.2011.37.0189

[197] Karschnia P, Parsons MW, Dietrich J. Pharmacologic management of cognitive impairment induced by cancer therapy. Lancet Oncol. 2019;20(2):e92–102. 30723041 10.1016/S1470-2045(18)30938-0

[198] Barton DL, Burger K, Novotny PJ, Fitch TR, Kohli S, Soori G, et al. The use of Ginkgo biloba for the prevention of chemotherapy-related cognitive dysfunction in women receiving adjuvant treatment for breast cancer, N00C9. Support Care Cancer. 2013;21(4):1185–92. 23150188 10.1007/s00520-012-1647-9 PMC3587364

[199] Treanor CJ, McMenamin UC, O'Neill RF, Cardwell CR, Clarke MJ, Cantwell M, et al. Non-pharmacological interventions for cognitive impairment due to systemic cancer treatment. Cochrane Database Syst Rev. 2016;2016(8):Cd011325. 10.1002/14651858.CD011325.pub2 PMC8734151 27529826

[200] Kirova YM, De Rycke Y, Gambotti L, Pierga JY, Asselain B, Fourquet A. Second malignancies after breast cancer: the impact of different treatment modalities. Br J Cancer. 2008;98(5):870–4. 18268495 10.1038/sj.bjc.6604241 PMC2266852

[201] Dong C, Chen L. Second malignancies after breast cancer: The impact of adjuvant therapy. Mol Clin Oncol. 2014;2(3):331–6. 24772296 10.3892/mco.2014.250 PMC3999127

